# Enhancing transmission type frame structures: A BBO algorithm-based integrated design approach

**DOI:** 10.1371/journal.pone.0300961

**Published:** 2024-05-17

**Authors:** Jian Yang, Zhiyong Yang, Yuhao Wang

**Affiliations:** 1 State Grid LeShan Power Supply Company, Sichuan, Leshan, China; 2 Shantou University, Sichuan, Chendu, China; Jamia Millia Islamia, INDIA

## Abstract

The stable and site-specific operation of transmission lines is a crucial safeguard for grid functionality. This study introduces a comprehensive optimization design method for transmission line crossing frame structures based on the Biogeography-Based Optimization (BBO) algorithm, which integrates size, shape, and topology optimization. By utilizing the BBO algorithm to optimize the truss structure’s design variables, the method ensures the structure’s economic and practical viability while enhancing its performance. The optimization process is validated through finite element analysis, confirming the optimized structure’s compliance with strength, stiffness, and stability requirements. The results demonstrate that the integrated design of size, shape, and topology optimization, as opposed to individual optimizations of size or shape and topology, yields the lightest structure mass and a maximum stress of 151.4 MPa under construction conditions. These findings also satisfy the criteria for strength, stiffness, and stability, verifying the method’s feasibility, effectiveness, and practicality. This approach surpasses traditional optimization methods, offering a more effective solution for complex structural optimization challenges, thereby enhancing the sustainable utilization of structures.

## Introduction

With economic growth, the pace of power grid construction has accelerated, leading to numerous overhead transmission lines traversing highways, railways, and other infrastructures [1–3]. Truss structures, commonly used for transmission spans, are extensively studied and applied in engineering structure optimization due to their computational and straightforward structural characteristics [4–6]. Truss structure optimization is categorized into size optimization, size and shape optimization, and size, shape, and topology optimization.

Size optimization, the most established method in truss structures, forms the foundation of engineering structure optimization. On the other hand, size, shape, and topology optimization, also known as layout optimization, represents a more advanced and challenging method involving various optimization design variables that can introduce coupling difficulties, complicating the achievement of precise and viable optimization outcomes [7, 8]. The surge in optimization design theory research and the widespread adoption of intelligent optimization algorithms in engineering design have heightened the demand for engineering structure optimization, making truss structure optimization a focal point of research.

In truss structure optimization, dimensional optimization focuses on the cross-sectional size of structural members, typically adhering to factory standards to fulfill standardization and economic criteria. Size and shape optimization, in contrast, involves both section size and structural shape variables, requiring the determination of node connections [9, 10]. The integration of discrete and logical variables in size, shape, and topology optimization presents challenges in addressing coupled optimization problems with diverse types of design variables.

In the realm of structural optimization algorithms, Di Trapani, F., et al., utilized genetic algorithms to address the optimization challenge of modifying shear-critical RC columns with steel jackets [11]. Pham, TD, et al., employed algorithms to rank each design within a swarm for the optimal design of precast beams, leveraging probabilistic natural selection [12]. Kaveh, A., et al., explored meta-heuristic algorithms for benchmark testing of steel frames and enhanced the design of 3D multilayered reinforced concrete (RC) structures using an advanced plasma generation optimization algorithm (IPGO) and applied the Lion Pride Optimization Algorithm (LPOA) to the optimal design of double-layered barrel vaults [13–15]. Daryan, AS, et al., introduced an innovative dolphin echolocation (DE) and bat optimization algorithm to develop a viable method for optimizing the design of steel plate shear wall frames [16]. Pan, W, proposed a comprehensive design process that integrates a flexible parametric model, an interdisciplinary evaluation criteria framework, and multi-objective optimization (MOO) with post-processing tools [17]. Dong, YR, et al., utilized algorithms to create a simulation testbed that elucidates the control law governing viscoelastic dampers’ impact on the seismic response of reinforced concrete frame structures [18]. Gong, J, et al., based their work on the seismic vulnerability analysis method (N-MASI-SFAM) and developed three analytical models to investigate the seismic vulnerability of transmission towers and lines under multi-angle seismic impacts [19, 20]. Zhao, GY, et al., conducted an experimental investigation into the ultimate bearing capacity of X-cross bracing in a 1000kV extra-high voltage steel tube transmission tower [21]. Liu, ZX, et al., merged spectrum representation with orthogonal decomposition, rooted in the coherence function matrix, to formulate a unified spectral decomposition expression [22]. Heiland, T, et al., utilized finite element methods to create a planar interaction model for a half-space and frame bridge, identifying the fundamental structure influenced by the first vertical intrinsic frequency to guide design optimization [23]. Lastly, Zhao, PJ, et al., introduced a novel approach to the design of girder arrangements in frame structures [24], while Huang, JD, et al., applied the durability time method for a precise and effective assessment of seismic performance [25]. These studies showcase the application and development of various algorithms in the field of structural optimization, providing a solid foundation for our further exploration of optimization algorithms.

Meanwhile, in terms of simulation analysis and performance studies, Jia, MM et al. analyzed the performance of BRBF connections with reduced beam sections and investigated the deformation capacity of ductile connections [26]. Sui, WN et al. conducted an experimental study on a steel frame structure system with a ratio of 1:2 under constant axial force and low circumferential horizontal reciprocating load [27]. Zhang, Q et al. proposed a stochastic subspace identification method for identifying tower modal parameters to study the effect of tower-line coupling on the frequency and vibration patterns [28]. Tan, Z et al. validated the finite element modeling method using the results of static loading tests and established numerical models for three unequal span combined beam-column assemblies [29]. Fei, QG et al. proposed a structural condition assessment method for transmission lines based on dynamic and stability analysis [30]. Li, TF et al. developed a remote cooperative hybrid test (RCHT) system based on the OpenFresco platform [31]. Sarassantika, IPE et al. evaluated the performance of a frame equipped with a new lever arm damper in support (LAD-Brace) [32]. Fan, W et al. performed a vibration analysis of a periodic multi-span transmission line system, analyzing its bandgap characteristics [33]. Wang, J et al. proposed a wind vulnerability framework with uncertainties in each line component, wind environment, and aerodynamic parameters of transmission towers [34]. Bai, JT et al. innovatively investigated the three-point bending collapse of DRTWT by combining internal and external topological descriptor functions for structural optimization while optimizing numerical algorithms to validate the frame model [35–37]. Gui, CY et al. proposed an integrated crashworthy design for a plastic frame model [38]. Çarbaş’s research demonstrates that the biogeography-based optimization algorithm excels in the optimal structural design of spatial steel frames over other metaheuristic algorithms. The results show that this algorithm surpasses others such as the adaptive firefly algorithm, teaching-learning-based optimization, artificial bee colony optimization, dynamic harmony search algorithm, and ant colony algorithm in the considered design examples [39]. Artar and Çarbaş investigated the optimal discrete sizing design of steel truss bridges using Teaching-Learning Based Optimization (TLBO) and Biogeography-Based Optimization (BBO) algorithms to minimize structural weights. The results show that the performance of these algorithms on both planar and spatial steel truss bridges outperforms the previously reported optimum designs obtained through other metaheuristics [40]. Tunca and Çarbaş investigated the optimization of a planar steel frame designed according to AISC-LRFD (American Institute of Steel Construction-Load and Resistance Factor Design) requirements using the biogeography-based optimization (BBO) algorithm. The results indicate that the BBO algorithm performs excellently in the optimization of planar steel frame designs [41]. Despite these successes, the BBO algorithm faces challenges and difficulties, particularly in dealing with highly nonlinear and complex design spaces where the convergence to global optima can be slow. The algorithm’s performance is also sensitive to its initial parameters and the setting of migration and mutation rates, which require careful tuning to avoid premature convergence or excessive computational time.

In view of the above, this paper builds upon various dimensional optimization algorithms and delves into the core aspects of topology optimization for truss structures. For planar truss structures, logical topology variables indicate the presence of rod connections between nodes. In contrast, for spatial truss structures, the pre-established arrangement of diagonal rods serves as logical topology variables. The optimization integrates discrete section and shape variables to refine the truss design’s size, shape, and topology. This truss design optimization encompasses size, shape, and topology by amalgamating discrete section and shape variables. Ultimately, the method’s feasibility and effectiveness in optimizing the truss structure’s size, shape, and topology are corroborated through the command flow in ANSYS and the optimization design program in MATLAB, facilitating a comprehensive optimization that combines both tools. This paper addresses the optimization design problem of traditional transmission line crossing frameworks and proposes a comprehensive optimization design method based on the Biogeography-Based Optimization (BBO) algorithm. Compared to existing optimization methods, the main innovations and contributions of this study are reflected in the following aspects:

1. Comprehensive Optimization Method: The study not only considers the size optimization of the beam structure but also integrates shape and topology optimization, achieving a holistic optimization of the crossing framework’s size, shape, and topology. This comprehensive optimization method enhances the structure’s performance more comprehensively while ensuring the design’s economic and practical viability.
2. Application of a Novel Algorithm: This paper is the first to apply the Biogeography-Based Optimization algorithm in the structural optimization design of transmission line crossing frameworks, demonstrating the algorithm’s effectiveness in complex structural optimization problems. The introduction of the BBO algorithm offers a new pathway for solving similar engineering structural optimization challenges.
3. Validation with Practical Engineering Applications: Through the command flow of the structural model written in ANSYS language and the optimization design program written in MATLAB language, this study validates the feasibility and effectiveness of the proposed optimization method. The approach is innovative theoretically and has excellent prospects for practical engineering applications.

## Cross-frame structure design

### Overview of the cross-frame project

The spanning frame serves as an overhead support for transmission lines when they cross roads, railroads, communication lines, power lines, and other obstacles. This section discusses a pioneering project that explores a new type of mobile lattice spanning frame designed for transmission lines exceeding 110KV. This frame is constructed from multiple standard sections, including the main pole, diagonal web pole, cross web pole, and connecting pole.

**Fig 1:**
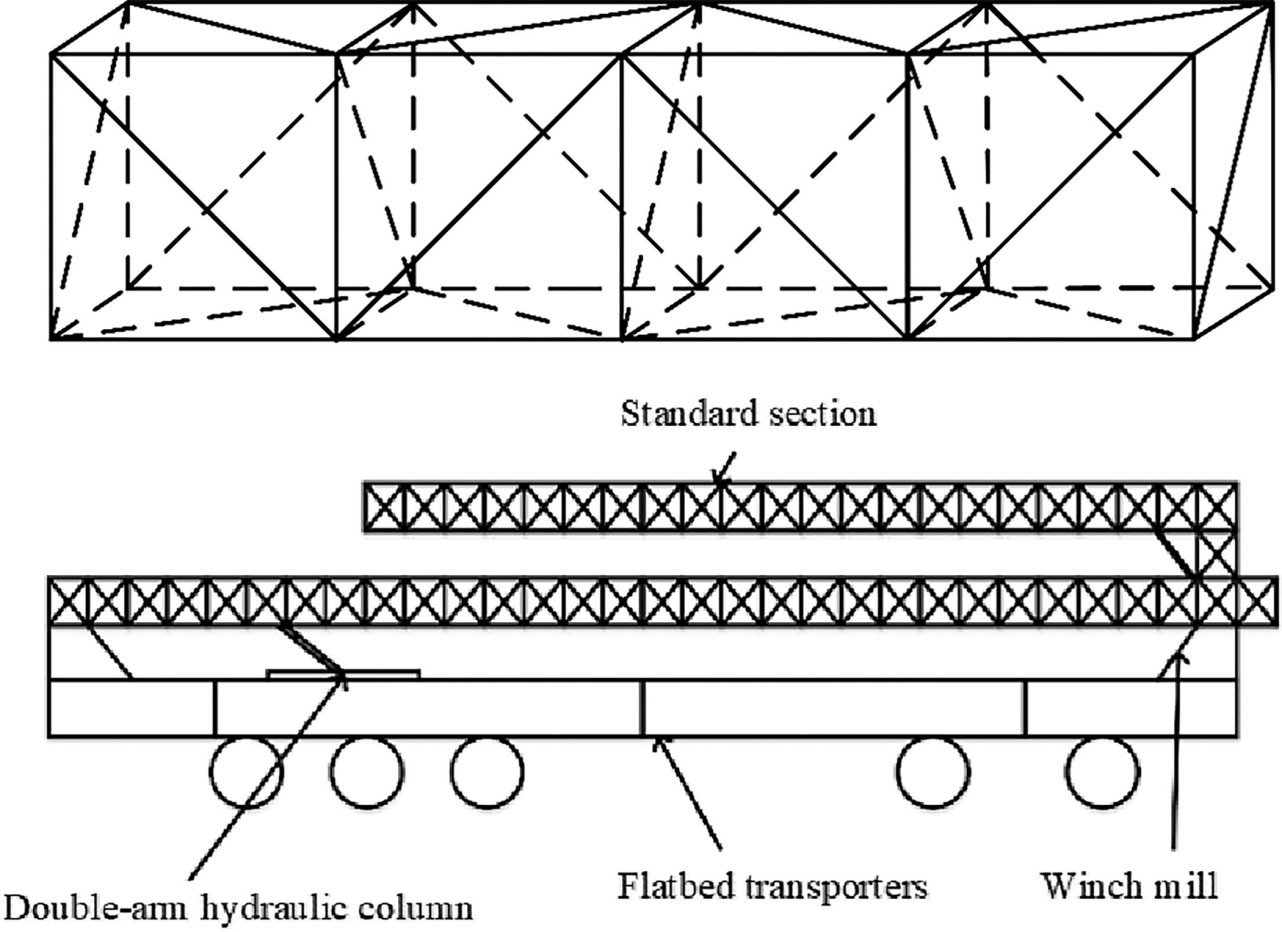
Standard section and transportation status diagram of crossing frame. Fig (a) Schematic diagram of the standard section of the cross-frame. Fig (b) Transportation status diagram.

The features of this new spanning frame are delineated in Table 1. It is composed of several standard sections and is equipped with a hydraulic power system, enabling rapid deployment and retraction of the structure. The frame’s modular design allows for flexible assembly and disassembly, adapting to various crossing heights and widths to meet the diverse requirements encountered at construction sites. Once assembled, the structure can be transported to the site on a vehicle, facilitating swift relocation and enhancing line construction efficiency, as illustrated in Fig 1.

**Table 1:** Parameter information of each element.

Rod classification	Elastic modulus Es[Mpa]	Mass density *ρ* [kg∙m^3^]	Quantity
Main column vertical bar	2.06×10^6^	7.85×10^3^	96
Main column crossbar	2.06×10^6^	7.85×10^3^	100
Main column diagonal web bar	2.06×10^6^	7.85×10^3^	96
Main column connecting bar	2.06×10^6^	7.85×10^3^	25
Boom vertical bar	2.06×10^6^	7.85×10^3^	64
Boom crossbar	2.06×10^6^	7.85×10^3^	68
Jib diagonal web bar	2.06×10^6^	7.85×10^3^	64
Boom linkage bar	2.06×10^6^	7.85×10^3^	17

On-site, depending on the span’s technical specifications and environmental conditions, one or two frames can be assembled. Ground brackets are adjusted to ensure the platform remains level and are anchored into the soil for stability. If necessary, the ground may undergo reinforcement. The frame’s main rod is positioned vertically using the hydraulic system, and the boom is elevated to align with the ground. To complete the setup, a guiding rope is installed between the boom’s sides, and a sealing net is established to create a conduit for the line.

### Engineering load condition design

According to the actual working condition of the span frame, now consider the span frame in working condition mainly bear the role of the structure self-weight, sealing network load and wind load and other loads. Referring to the crane design specification, the calculation formula of wind load is:

$$\left\{ \begin{array}{c} P_{w}=CpA\\ p=0.625v_{s}^{2} \end{array}\right.$$ (1)

In Expression (1), *P*_*w*_ is the maximum wind load acting on the working state of the span frame, the unit is *N*; *p* is the maximum calculated wind pressure of the normal working state, the unit is 2N/m; *v*_*s*_ is the calculated wind speed, the unit is *m/s*; A is the solid windward area of the span frame structure perpendicular to the wind direction, the unit is *m*^*2*^; *C* is the wind coefficient, the wind coefficient of this structure is 1.7 (1+*η*), where the wind blocking discount The value of *η* is determined by the filling rate of the windward side of the span frame structure and the spacing ratio. The spacing ratio is *a/B*, *a* is the distance between the relative surfaces of the two components, *B* is the width of the windward surface of the component; the structural windward surface filling rate *φ* is:

$$\varphi =\frac{A^{*}}{A_{0}^{*}}=\sum_{i=1}^{n}\frac{l_{i}\times b_{i}}{L\times B}$$ (2)

In Expression (2), A* is the area of the solid part and $A_{0}^{*}$ is the area of the profile. The length *l*_*i*_ of a single member is taken as the central spacing of adjacent nodes, *b*_*i*_ is the section width of the windward side of the member section, and *L* is the height of the standard section of the lattice type.

Since the cross-sectional size and length of the span frame are set as variables, and the magnitude of wind load is related to the cross-sectional size and length of the bars, the magnitude of wind load is also variable. The wind load is converted into a concentrated load and loaded on the main column and the arm structure equally; and the sealing network load is converted into a concentrated load and loaded on the arm structure at a spacing of 1m.

### Overview of the cross-frame optimization model

Depending on the varying levels and complexities of design requirements, optimization of the span frame structure can be categorized into three types: size optimization, size and shape optimization, and size, shape, and topology optimization. In size optimization, the section size is the primary design variable, with fixed coordinate positions and rod connections. For size and shape optimization, both section size and coordinate position variables are optimized, while rod connections remain unchanged. In size, shape, and topology optimization, the design variables include cross-sectional dimensions, structural coordinate positions, and rod connections. This paper synthesizes these approaches into a comprehensive method for size, shape, and topology optimization of span frame structures, drawing on biogeography-based principles.

Optimization model;

The mathematical model for the optimization of the cross-frame structure is simplified as:

$$\min\;W=\sum_{j=1}^{n}\rho_{j}A_{j}L_{j}(x)\;(j=1,2,\ldots n)$$ (3)

In Expression(3), $s.t.\;g_{e}(A_{j},T_{j},L_{j}(x))\leq 0\;(e=1,2,\ldots NC);A_{i}\in S=\{S_{1},\ldots S_{ND}\},i=1,2,\ldots NP;x=\{x_{1},\ldots x_{NS}\},x_{min}\leq x_{m}\leq x_{max},m=1,2,\ldots NS;T_{k}=\{1,2,3,4\},k=1,2,\ldots NG$. *W* is the total mass of the span frame structure; *L*_*j*_ and *ρ*_*j*_ are the length and density of the span frame bars, respectively; *A*_*j*_ is the cross-sectional area of each bar of the span frame; *x* is the structural shape design variable, selected as the coordinates of the key nodes; n is the total number of structural bars; *g*_*e*_ is the constraint function, selected as the structural stiffness, strength constraint and stability constraint of the bars; NC is the number of constraint functions; *A*_*i*_ is the bar section design variables; NP is the number of section size design variables; S is the discrete domain of cross-frame structural rod section variables; ND is the number of discrete section models; *x*_*min*_ and *x*_*max*_ are the upper and lower limits of node coordinates *x*_*k*_, respectively; m is the number of shape design variables; NS is the number of structural shape control parameters; *T*_*k*_ is the topology design variables of diagonal web rod layout form, and NG is the number of topology variables.

The optimization model for span frame structure can be represented as a mathematical model for size, size and shape, and size shape and topology optimization problems, respectively. Size optimization: when the shape design variable *x* and the topology design variable *T*_*k*_ in Expression (3) are fixed, the mathematical model is expressed as the span frame structure size optimization design model; when only the topology design variable *T*_*k*_ in in Expression (3) is fixed, the mathematical model is expressed as the span frame structure size and shape optimization design model; when all the design variables in Expression (3) are variable, the mathematical model When all the design variables in Expression (3) are variable, the mathematical model is expressed as the optimized design model of span frame structure size, shape and topology.

Penalty function method.

If the weight of the span frame structure is used as the objective function, the location of the initial population generated by the optimization algorithm may not satisfy the constraints even if it can satisfy the constraints, but the new population generated after the migration, mutation and removal operations may not satisfy the constraints, so this chapter also uses the penalty function to deal with the constraints. The basic idea of the penalty function method is to transform the constrained optimization problem into an unconstrained optimization problem to solve. The penalty function is a function obtained by combining the objective function and the constraint function, and the conditions in Expression (3) can be defined for the constrained optimization problem.

The following penalty function can be defined:

$$F(\underset{x}{\to })=W+K\sum_{i=1}^{NC}g_{e}^{2}(A_{j},T_{j},L_{j}(x))$$ (4)

In Expression (4), *K* is the penalty function parameter (penalty factor), which indicates the magnitude of the penalty strength. The value of the penalty function parameter *K* is determined in order to convert the constrained problem into an unconstrained problem and achieve the optimal solution of the original objective function. Choosing too large a *K* value can speed up the fast convergence of the algorithm, but too large a *K* value can lead to difficulties in solving the corresponding unconstrained problem accurately, and the *K* value can be obtained by debugging the algorithm to take 10^10^ to achieve the optimal solution of the original objective function.

**Fig 2:**
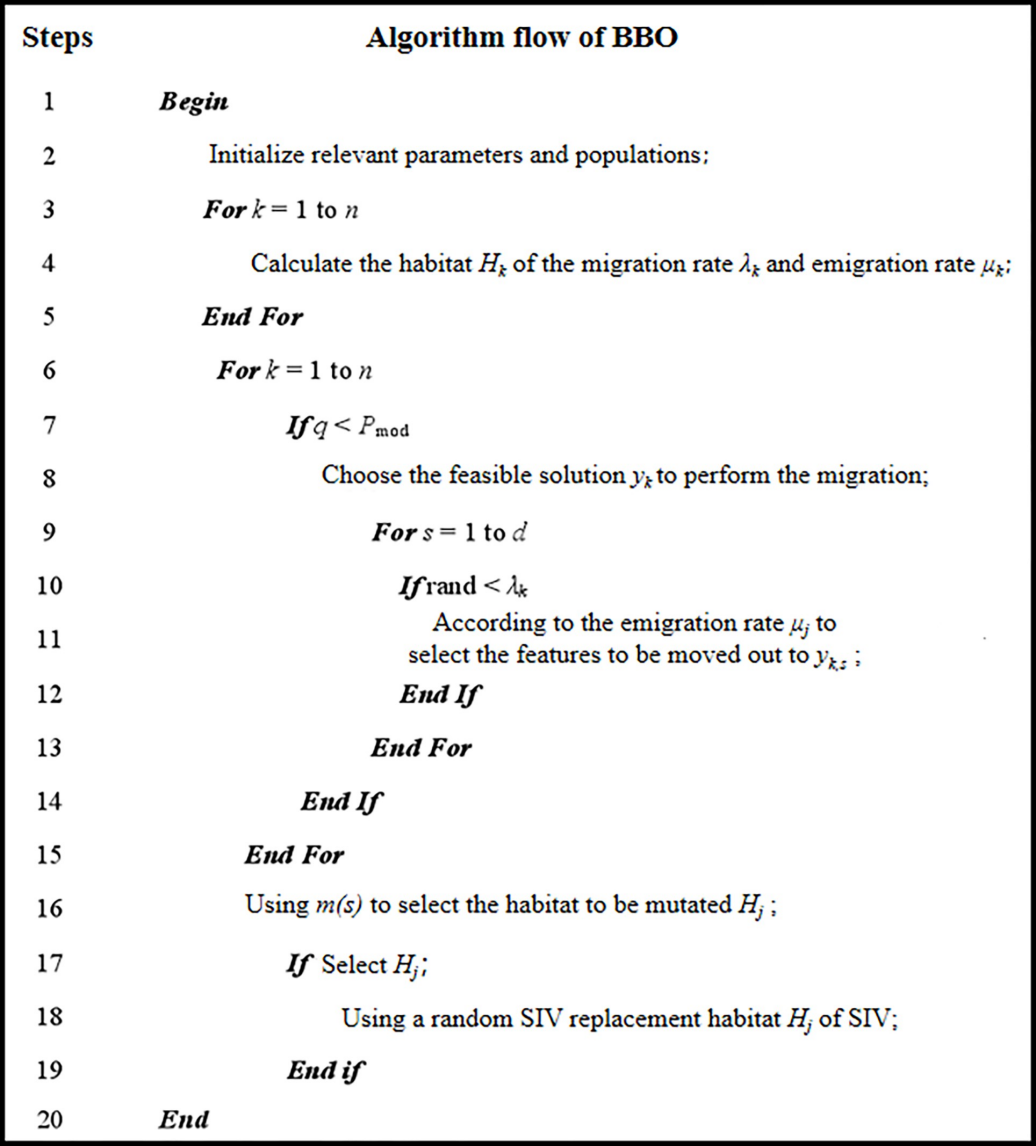
Algorithm flow of BBO.

The optimal solution using penalty function can be established based on the BBO algorithm with the computational setup flowchart shown in Fig 2. below, where the variation of habitat can be executed based on the chance of the total number of species in the habitat for the purpose of finding the best. Assume that the population size is *n* and the population *Y* contains n feasible solutions *y*_k_, *k* ∈ [1,*n*]. *H*_*k*_ is the habitat of the BBO algorithm; the migration rate *λ*_*k*_ and the migration rate *μ*_*k*_; SIV is the adaptation index variable; the variation rate satisfies *m(s) = m*_*max*_, where *m*_*max*_ is a predetermined maximum variation rate; *P*_*s*_ is the probability that the number of species is *s*; *P*_*max*_ *= max{P*_*s*_*}*.

The proposed BBO algorithm-based optimization design method for cross-frame structures involves the following steps:

Step 1: Develop a finite element model of the structure, incorporating material properties, nodal constraints, and loads. Parameterize the cross-sectional size variables, structural shape variables, and topological variables of the members. Configure the BBO algorithm parameters, including the maximum immigration rate, maximum emigration rate, maximum mutation rate, global migration rate, and the number of elite individuals retained.

Step 2: Initialize the probability of species quantity in habitats and the suitability vectors of habitats. Each vector corresponds to a potential feasible solution, allowing the initial individual variables to be randomly generated within the search space.

Step 3: Conduct a structural analysis of the initial population, calculating structural form variables (such as stress and nodal displacement of each member) and fitness values for each individual. Apply penalty values to individuals violating constraints and select the optimal and elite individuals.

Step 4: Perform a clearing operation, comparing fitness vectors one by one. When two identical fitness vectors are found, a new fitness vector is randomly generated from the search space to replace the duplicate one.

Step 5: Compute the habitat suitability, arranging habitats in descending order of suitability.

Step 6: Save the optimal solution. If the termination condition is met, stop the optimization process and output the optimization results; otherwise, proceed to Step 7.

Step 7: Execute the migration operation, calculating the species quantity for each habitat and determining the immigration and emigration rates to assess whether habitats can undergo immigration operations.

Step 8: Update the probability of species quantity in habitats and then calculate the mutation rate of habitats, sequentially determining whether each characteristic component of the habitats mutates. Return to Step 4 and begin a new cycle.

### Cross-frame optimization model construction

Considering the diagonal web rods in the span frame structure as auxiliary materials, there are numerous and variable arrangement forms, and its layout form plays an important influence on the economy and mechanical performance of the whole span frame. In this section, the layout form of diagonal webs is taken as the topological design variable, and the layout form that meets the engineering requirements is preset in advance as the variable space, which can ensure the reasonable layout of the optimized bars.

By means of structural size, shape and topology optimization, the constraints and design variables of the span frame structure are carried out with the minimum structural weight of the span frame as a whole as an objective function as shown below.

Constraint conditions;

For size, shape and topology optimization of the span frame, the allowable stress of all bars in the structure is 156.6 MPa; the displacement constraint is that the deformation at the end of the boom cannot exceed 34.28 mm, and the deformation at the connection between the main column and the boom cannot exceed 241.2 mm; the length-to-thin ratio of the main column vertical rod and the arm vertical rod is constrained to 150, the length-to-thin ratio of the main column crossbar to the boom crossbar is constrained to 180, and the length-to-thin ratio of the main column diagonal web, main column link, arm brace diagonal web, and arm brace link is constrained to 200.

Design variables;

The overall span frame is formed by the same standard section, the cross-sectional area of the standard section, node position and diagonal web arrangement type are the key factors for the optimization of the overall span frame, so the above three key factors as variables, the weight of the span frame as the objective function, set the variable space in line with the actual working conditions and specification requirements of the project, the span frame structure optimization.

Dimensional variables;

The same dimensional variables as those used to perform dimensional optimization and size and shape optimization are used for a total of 8 dimensional variables and are denoted by *A*_*i*_ (i = 1,2,3… 8).The discrete section libraries for the main column and boom diagonal webs and connecting rods are shown in Table 2, the discrete section libraries for the boom vertical rods and cross rods are shown in Table 3, and the discrete section libraries for the main column vertical rods and cross rods are shown in Table 4. The section libraries for each rod are equilateral *L* sections, each with 20 profiles available for searching, and the design variables for dimensional optimization are {*A*_1_,…*A*_8_}.

**Table 2:** Discrete section library of diagonal web member and connecting rod.

Number	Model[mm]	Area[mm^2^]	Number	Model[mm]	Area[mm^2^]
1	L25×25×3.0	143.9	11	L45×45×3.0	265.9
2	L25×25×4.0	185.9	12	L45×45×4.0	348.6
3	L30×30×3.0	174.9	13	L45×45×5.0	428.2
4	L30×30×4.0	227.6	14	L45×45×6.0	507.6
5	L36×36×3.0	210.9	15	L50×50×3.0	297.1
6	L36×36×4.0	275.6	16	L50×50×4.0	389.7
7	L36×36×5.0	338.2	17	L50×50×5.0	480.3
8	L40×40×3.0	235.9	18	L50×50×6.0	568.8
9	L40×40×4.0	308.6	19	L56×56×3.0	334.3
10	L40×40×5.0	379.1	20	L56×56×4.0	439.0

**Table 3:** Discrete section library of boom vertical bar and cross bar.

Number	Model[mm]	Area[mm^2^]	Number	Model[mm]	Area[mm^2^]
11	L45×45×3.0	265.9	21	L56×56×5.0	541.5
12	L45×45×4.0	348.6	22	L56×56×8.0	836.7
13	L45×45×5.0	429.2	23	L63×63×4.0	497.8
14	L45×45×6.0	507.6	24	L63×63×5.0	614.3
15	L50×50×3.0	297.1	25	L63×63×6.0	728.8
16	L50×50×4.0	389.7	26	L63×63×8.0	951.5
17	L50×50×5.0	480.3	27	L70×70×4.0	557.0
18	L50×50×6.0	568.8	28	L70×70×5.0	687.5
19	L56×56×3.0	334.3	29	L70×70×6.0	816.0
20	L56×56×4.0	439.0	30	L70×70×7.0	942.4

**Table 4:** Discrete section library of main column vertical bar and cross bar.

Number	Model[mm]	Area[mm^2^]	Number	Model[mm]	Area[mm^2^]
21	L56×56×5.0	541.5	31	L70×70×8.0	1066.7
22	L56×56×8.0	836.7	32	L75×75×5.0	736.7
23	L63×63×4.0	497.8	33	L75×75×6.0	879.7
24	L63×63×5.0	614.3	34	L75×75×7.0	1016.0
25	L63×63×6.0	728.8	35	L75×75×8.0	1150.3
26	L63×63×8.0	951.5	36	L80×80×5.0	791.2
27	L70×70×4.0	557.0	37	L80×80×6.0	939.7
28	L70×70×5.0	687.5	38	L80×80×7.0	1086.0
29	L70×70×6.0	816.0	39	L80×80×8.0	1230.3
30	L70×70×7.0	942.4	40	L80×80×10	1512.6

### Shape variables

The four nodal coordinates in the *X-Z* plane are applied to the variables *x1*, *x2*, which denote the length of the main column cross web due to the variation of the nodal coordinates in the *X* and *Y* directions, and *x3* denotes the length of the boom cross web due to the variation of the nodal coordinates in the *Z* direction. The design domain of shape variables is *x1*∈[1000,1300](mm), *x2*∈[1000,1300](mm), and *x3*∈[1000,1300](mm), and the three shape design variables are searched in the design domain with 10 mm as discrete values. The shape variables are denoted by {*x1*, *x2*, *x3*}.

### Topological variables

Based on engineering experience and mechanics, a diagonal web on each surface is the most common and best mechanical arrangement in traditional lattice structures. Now the span frame is divided into two main parts: main column and boom, and different diagonal web topology types are established in advance.

**Fig 3:**
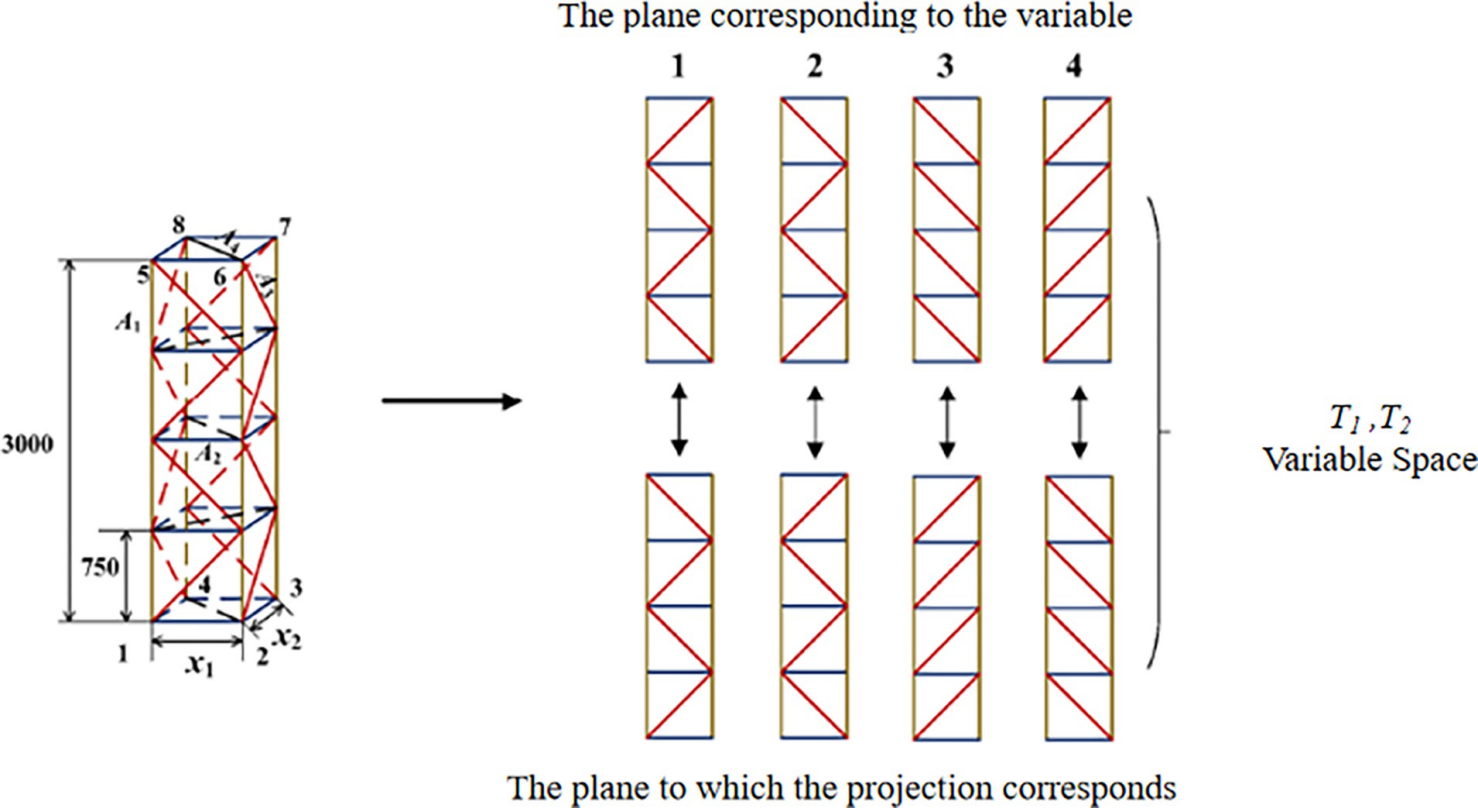
Schematic diagram of design variables for main column of crossing frame.

**Fig 4:**
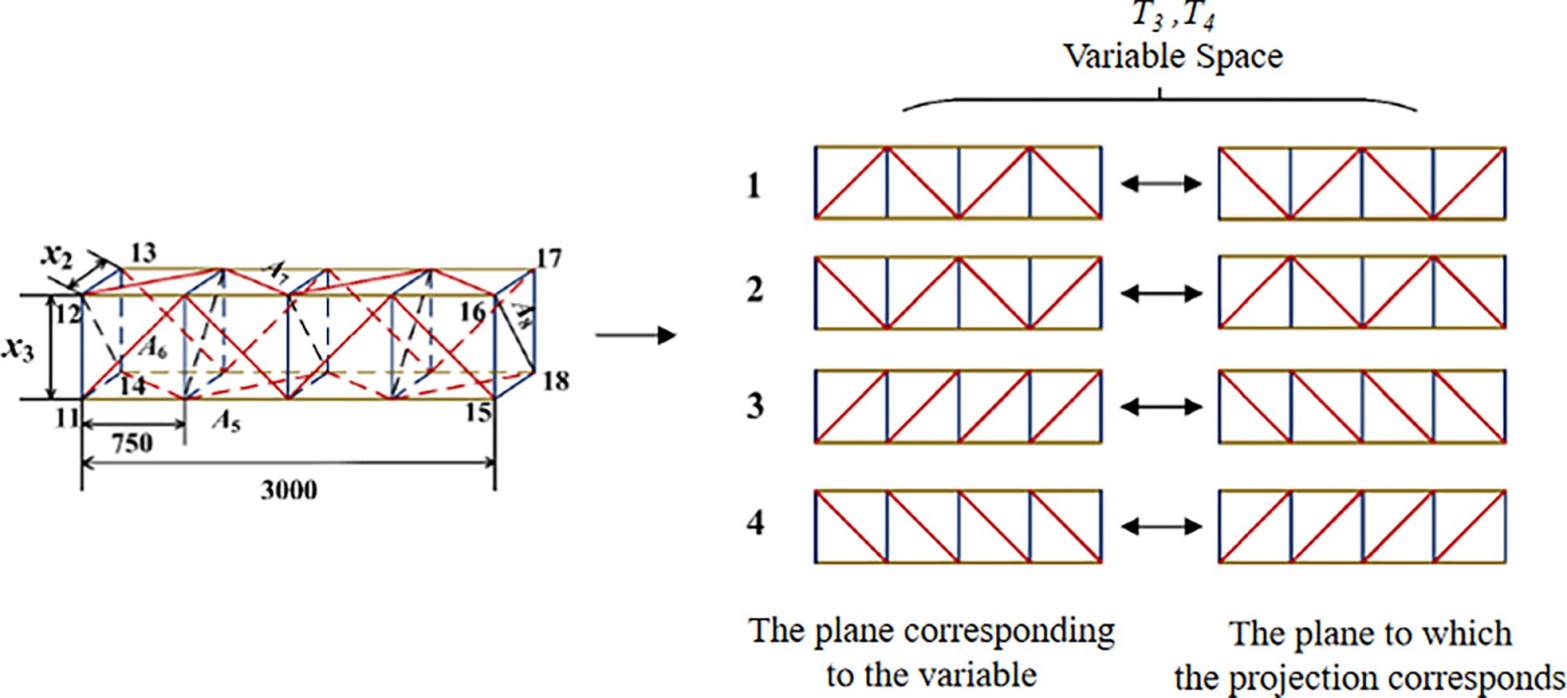
Schematic diagram of design variables of crossing frame.

Logical topological variables (*T1*, *T2*): if the form of the diagonal web arrangement for one side (example 1-2-5-6) is predetermined in advance, the form of the diagonal web arrangement for the plane parallel to it (3-4-7-8) is determined, and if the form of the diagonal web arrangement for one side (example 1-4-5-8) is predetermined in advance, the form of the diagonal web arrangement for the plane parallel to it (2-3-6-7) is determined, i.e. when the form of the diagonal web arrangement for the plane (1-2-5-8) is 1, the form of the diagonal web arrangement for the plane (3-4-7-8) is determined, as shown in Fig 3. (1-2-5-8) is 1, the layout of the plane (3-4-7-8) is 2, as shown in Fig 4.

Logical topological variables (T3, T4): if the form of the diagonal web arrangement of one side (11-12-15-16) is preset in advance, the form of the diagonal web arrangement of the plane (13-14-17-18) parallel to it is determined, and if the form of the diagonal web arrangement of one side (example 11-14-15-18) is preset in advance, the form of the diagonal web arrangement of the plane (12-13-16-17) parallel to it is determined. The form of the diagonal rod arrangement is determined, i.e., when the diagonal rod arrangement of the plane (11-12-15-18) is 1, the arrangement of the plane (13-14-17-18) is 2, as shown in Fig 4.

The design domain of topological variables is divided into four kinds of interlaced and parallel represented by 1, 2, 3 and 4, and the topological variables are expressed as *T*_*i*_ (*i* = 1,2,3,4), so there are 44 = 256 combinations of forms. The form of diagonal web rod arrangement corresponding to each plane is searched in the topological space and can be combined in any way. The design variables of the size, shape and topology optimization process are *x* = {*A*_1_,…*A*_8_, *x*_1_,…*x*_3_, *T*_1_,…*T*_4_}.

Optimization model;

The optimization model of lattice span frame structure size, shape and topology is:

$$\min\;W=\sum_{j=1}^{n}\rho_{j}A_{j}L_{j}(x)\;(j=1,2,\ldots n)$$

$$s.t.\;g_{1}(A_{j},L_{j}(x),T_{k})-156.6\leq 0$$

$$g_{2}(A_{j},L_{j}(x),T_{k})-34.28\leq 0,g_{3}(A_{j},L_{j}(x),T_{k})-241.2\leq 0,$$

$$g_{4}(A_{j},L_{j}(x),T_{k})-150\leq 0,g_{5}(A_{j},L_{j}(x),T_{k})-180\leq 0$$

$$g_{6}(A_{j},L_{j}(x),T_{k})-200\leq 0,g_{7}(A_{j},L_{j}(x),T_{k})-200\leq 0$$

$$A_{i}\in S=\{S_{1},S_{2}\ldots S_{20}\}\;(i=1,2,\ldots ,8)$$

$$x_{m}\in [1000,1010,1020,\ldots ,1290,1300]\;(m=1,2,3)$$

$$T_{k}=\{1,2,3,4\}\;(k=1,2,3,4)$$ (5)

In Expression (5), *T*_*k*_ is the topological design variable for the diagonal web arrangement form.

The parameters for setting the BBO algorithm when the span frame structure is optimized for size, shape and topology are shown in Table 5.

**Table 5:** Control parameters for size, shape and topology optimization of crossing frame.

**BBO Parameters**	**Number of populations**	**Number of iterations**	**Migration rate**	**Emigration rate**	**Maximum mutation rate**	**P_mod_**
	50	1000	1	1	0.01	1

Optimization results.

**Fig 5:**
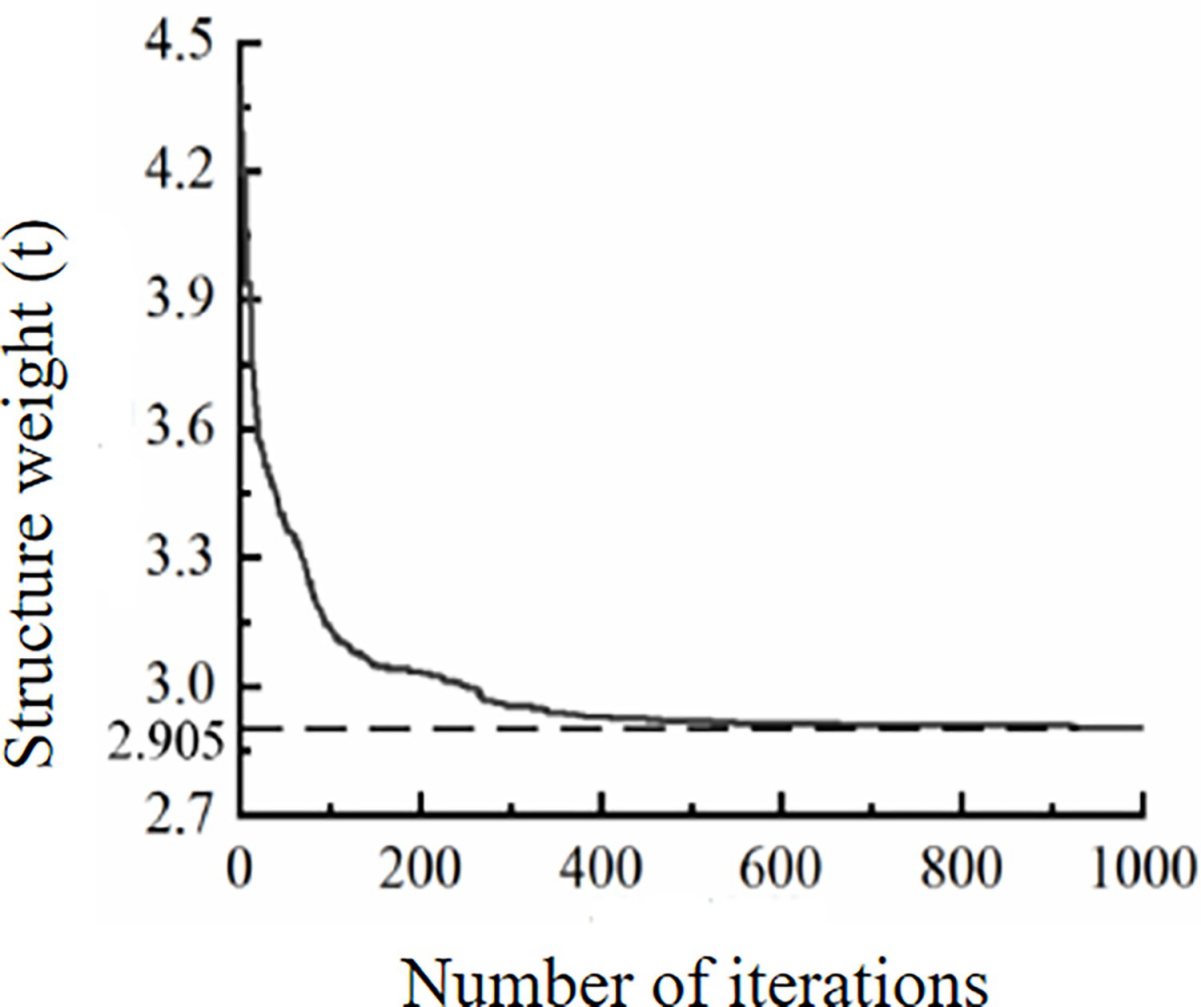
The size, shape and topology optimization iterative diagram of crossing frame.

After the optimization calculation, the iterative process of size, shape and topology optimization under the continuous iteration of biogeographic optimization algorithm, after 1000 iterations, is shown in Fig 5, and the optimization results are shown in Table 6., and the best optimization effect of span frame weight is 2.905t.

**Table 6:** The size, shape and topology optimization results of the crossing frame.

**Dimensional Variables**	*A*_1_	*A*_2_	*A*_3_	*A*_4_
**Model [mm]**	*L*63×63×5.0	*L*56×56×5.0	*L*40×40×4.0	*L*36×36×5.0
**Area [mm** ^**2**^ **]**	614.3	541.5	308.6	338.2
**Cross-section number**	24	21	9	7
**Shape variables**	*A*_5_	*A*_6_	*A*_7_	*A*_8_
**Model [mm]**	*L*45×45×6.0	*L*45×45×3.0	*L*36×36×4.0	*L*36×36×3.0
**Area [mm** ^**2**^ **]**	507.6	265.9	275.6	210.9
**Cross-section number**	14	11	6	5
**Shape variables**	*x*_1_ *x*_2_ *x*_3_			
**Rod length[mm]**	1010 1100 1000			
**Topological variables**	*T*_1_	*T*_2_	*T*_3_	*T*_4_
**Optimal scheduling**	1	1	4	3

**Fig 6:**
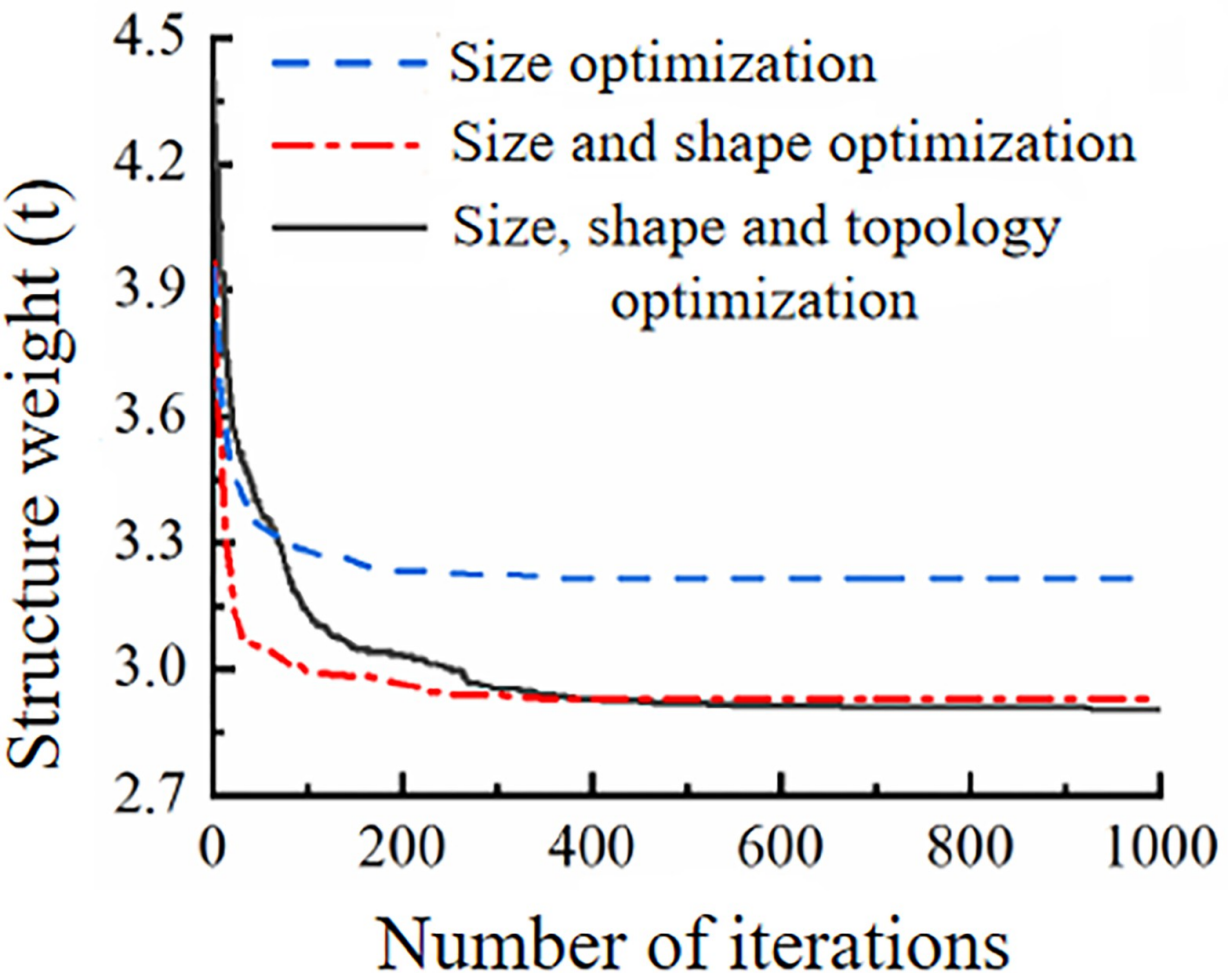
Comparison of three optimization processes.

The same method can be used to optimize size, size and shape separately. The iterative process of size optimization, size and shape optimization, and size, shape and topology optimization are compared in Fig 6. The optimization effect of size, shape and topology optimization is also improved compared with the optimization effect of size and shape optimization.

## Cross-frame structure optimization verification

### Loading conditions

#### Sealing net load

The capping net, woven with insulated nylon rope, consists of lattice rope, side rope, and horizontal net rope. In the context of this paper’s span frame, when the running line enters an accident state, it first impacts the outside of the span frame’s arm. After the line falls, the impact kinetic energy is significantly reduced or dissipated; thus, the capping net load calculation only considers the static load post-fall.

The side rope, made of polyester, has a radius of 12 mm (wire density is 0.080 kg/m), while the grid rope, also polyester, has a radius of 6 mm (wire density is 0.020 kg/m). For the optimized cross-frame structure, a single standard section on the boom measures 1010 mm by 1100 mm by 3000 mm (length by width by height). The x-y plane dimensions of a single cross-frame boom are 1100 mm by 3000 mm. The capping net’s self-weight, totaling 1100 kg, is distributed as a concentrated load along the edge and grid ropes on the boom. Consequently, a nodal load of 29.4 N is applied to each node under working conditions.

#### Wind load

Open-air work of the span frame should consider the effect of wind load. It is assumed that the wind load is the static load acting along the most unfavorable horizontal direction of the span frame, and the calculated wind pressure value is obtained according to the span frame type.

Calculation of wind pressure;

$$p=0.625v_{s}^{2}$$ (6)

In Expression (6): *p* is the calculated wind pressure in N/m2; *v*_*s*_ is the calculated wind speed in m/s.

Wind load calculation;

The working state wind load acting on the span frame is calculated according to the following two cases.

When the wind direction is perpendicular to the longitudinal axis of the member or the surface of the frame, the wind load along this wind direction is calculated by the following formula;

$$\left\{ \begin{array}{c} P_{w1}=Cp_{1}A\\ P_{w2}=Cp_{2}A \end{array}\right.$$ (7)

In Expression (7): A is the solid windward area of the span frame member perpendicular to the wind direction, the unit is m^2^; *P*_*w*1_ is the normal wind load acting on the span frame in working condition, the unit is N; *P*_*w*2_ is the maximum wind load acting on the span frame in working condition, the unit is N; according to the different calculation contents, *p*_1_ is 90 in working condition, *p*_2_ is 500 in high wind condition; *C* is the wind coefficient; this The wind coefficient of the structure is 1.7(1+*η*), in which the value of the wind blocking reduction factor *η* is determined by the filling rate of the windward side of the structure and the spacing ratio.

*A* is the solid windward area of the span frame member perpendicular to the wind direction in m^2^, which is equal to the profile area *A*_0_ of the windward side of the member multiplied by the filling rate *φ* of the windward side of the structure, that is, *A* = *A*_0_ *φ*; the spacing ratio is *a/B*, a is the distance between the relative surfaces of the two pieces of the member, *B* is the width of the windward side of the member; the structural windward side filling rate *φ* is:

$$\varphi =\frac{A^{*}}{A_{0}^{*}}=\sum_{i=1}^{n}\frac{l_{i}\times b_{i}}{L\times B}$$ (8)

**Fig 7:**
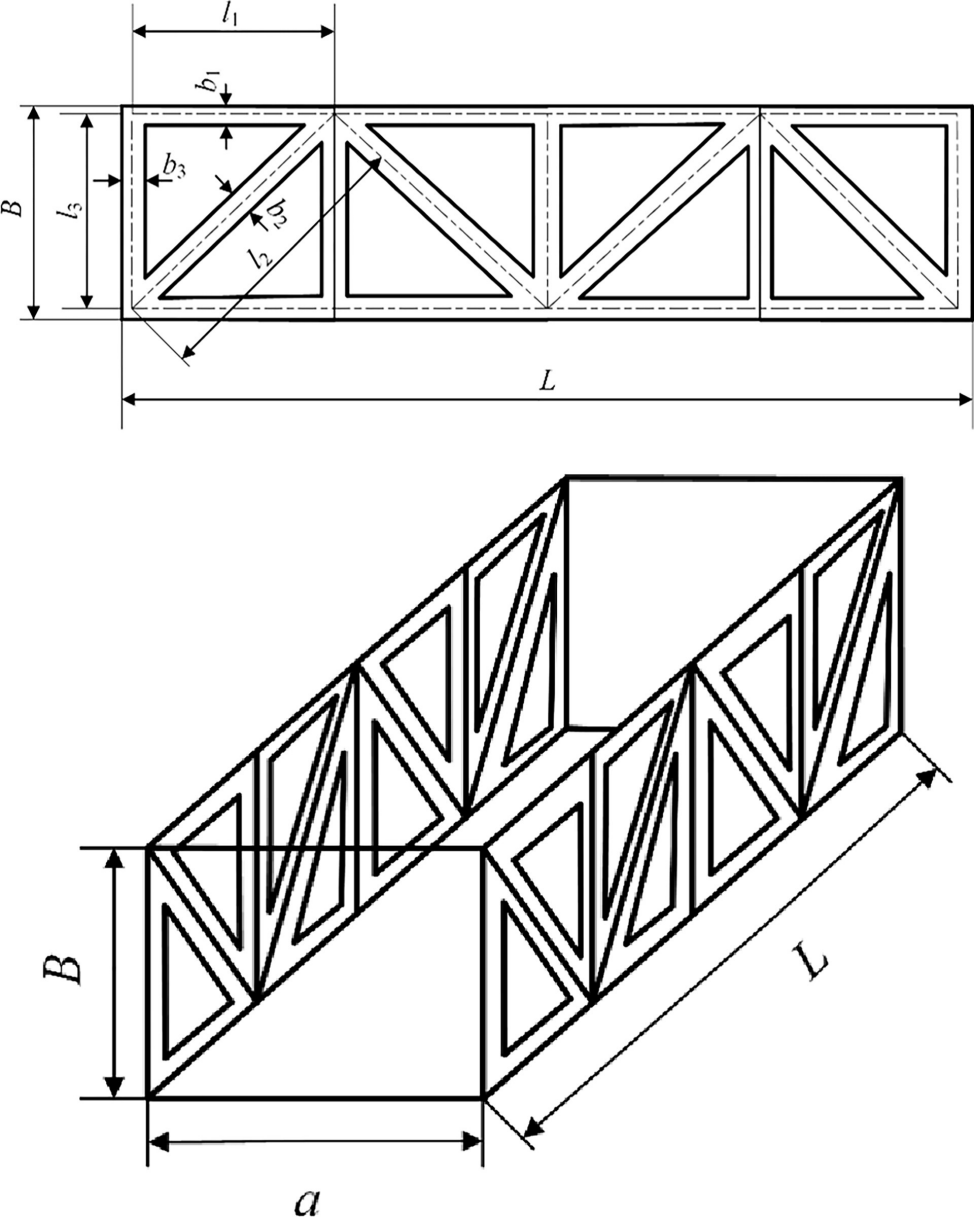
Definition of wind coefficient in calculation. Fig (a) Structural windward side. Fig (b) Interval ratio.

In Eq (8), A* is the area of the solid part, and $A_{0}^{*}$ is the area of the profile. The length *l*_*i*_ of a single member is taken as the center spacing of adjacent nodes, *b*_*i*_ is the cross-sectional width of the windward side of the member section, and *L* is the height of the lattice type standard section. The solid part area and contour area of each part of the span frame in each direction are obtained according to the size, shape and topology optimization results of the bar cross-sectional dimensions and shape dimensions, see Fig 7.

When the wind direction is at an angle with the longitudinal axis of the member or the surface of the frame, the wind load along this wind direction is calculated by the following formula.

$$\left\{ \begin{array}{c} P_{w1}=Cp_{1}A{sin}^{2}\theta \\ P_{w2}=Cp_{2}A{sin}^{2}\theta \end{array}\right.$$ (9)

In Expression (9), *P*_*w*1_, *P*_*w*2_, *p*_1_, *p*_2_, *C* are the same as Expression (7); *A* is the frontal wind area of the member parallel to the longitudinal axis, in square meters (m2); *θ* is the angle between the wind direction and the longitudinal axis of the member or the surface of the frame (*θ* <90°), in degrees (°).

According to the optimized cross-sectional dimensions of the bars, the wind load parameters of the lattice span frame can be obtained as shown in Table 7.

**Table 7:** Wind load parameters of lattice crossing frame.

Parameters	A*/*m*^2^	$A_{0}^{*}/m^{2}$	*φ*	Interval ratio	*η*	*C*
X-Z side of main column	5.172	18.18	0.28	1	0.52	2.584
Y-Z side of main column	5.394	19.8	0.27	1	0.53	2.601
Jib X-Z side	2.700	12.00	0.23	1	0.57	2.669
Jib X-Y side	2.836	13.20	0.21	1	0.60	2.720

## Calculation of working conditions

The span frame primarily supports the self-weight load, sealing network load, and wind load. The wind load is categorized into normal working wind load (at a wind speed of 15.5 m/s) and extreme wind load (at a wind speed of 30 m/s). The span frame structure is designed to ensure structural safety under high wind conditions, prevent overturning accidents, and maintain acceptable deformation under normal working conditions to meet the requirements of span release construction.

The construction assembly load combination consists of self-weight load, sealing network load, wind load, and other fundamental loads. The load combination is calculated to determine if the frame’s strength, stiffness, and stability meet engineering standards. The working frame line load combination includes the construction assembly load combined with the additional sealing net load. Since wind can damage the sealing net, it is considered retracted in high wind scenarios, resulting in a wind load combination of the original self-weight load plus the ultimate wind load. This combination primarily verifies the span frame’s overall structural stability and wind safety.

Based on the actual load-bearing scenario of the new lattice span, nine distinct load combinations are designed. The specific calculation working conditions for these combinations are detailed in Table 8.

**Table 8:** Working condition arrangement.

Name of working condition	Work condition classification	Working load
Working condition 1	Construction work conditions (Single spanning frame)	15.5m/s wind(+X direction)+ Self-weight
Working condition 2		15.5m/s wind(+Y direction)+ Self-weight
Working condition 3		15.5m/s wind(+45° direction)+ Self-weight
Working condition 4	Working conditions (Seal the net after docking)	15.5m/s wind(+X direction)+ Blocking the net
Working condition 5		15.5m/s wind(+Y direction)+ Blocking the net
Working condition 6		15.5m/s wind(+45° direction)+ Blocking the net
Working condition 7	High wind conditions (High wind condition after docking)	30m/s wind(+X direction)+ Self-weight
Working condition 8		30m/s wind(+Y direction)+ Self-weight
Working condition 9		30m/s wind(+45° direction)+ Self-weight

### Cross-frame strength analysis

**Fig 8:**
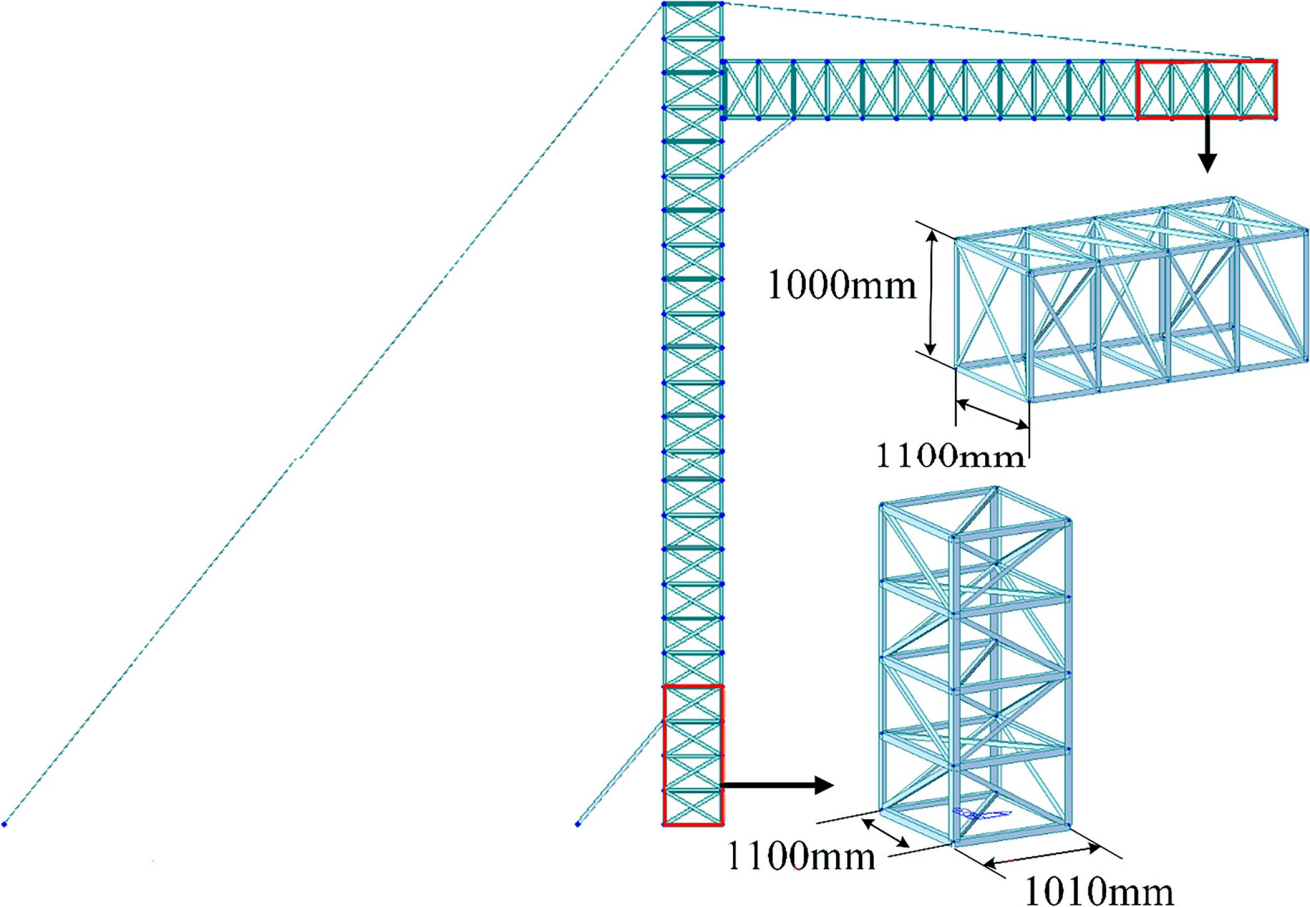
Optimized model size of crossing frame.

After the optimization algorithm to optimize the size, shape and topology of the lattice spanning frame, the lightest weight, the most material-saving rod cross-section and structural shape, and the most reasonable form of rod arrangement were obtained, and the finite element model of the spanning frame before docking is shown in Fig 8.

The strength analysis of the optimized span frame model is carried out under the combined load action of each working condition. In this section, the results of stress calculation and analysis of the span frame in the construction assembly condition, the working condition of the line, and the extreme wind condition are given. The finite element analysis is carried out sequentially according to the load conditions and the working arrangement. Considering the length and the large area of the windward side in the +Y direction and 45° direction, the following stress clouds in the +Y direction and 45° direction are listed for each condition.

**Fig 9:**
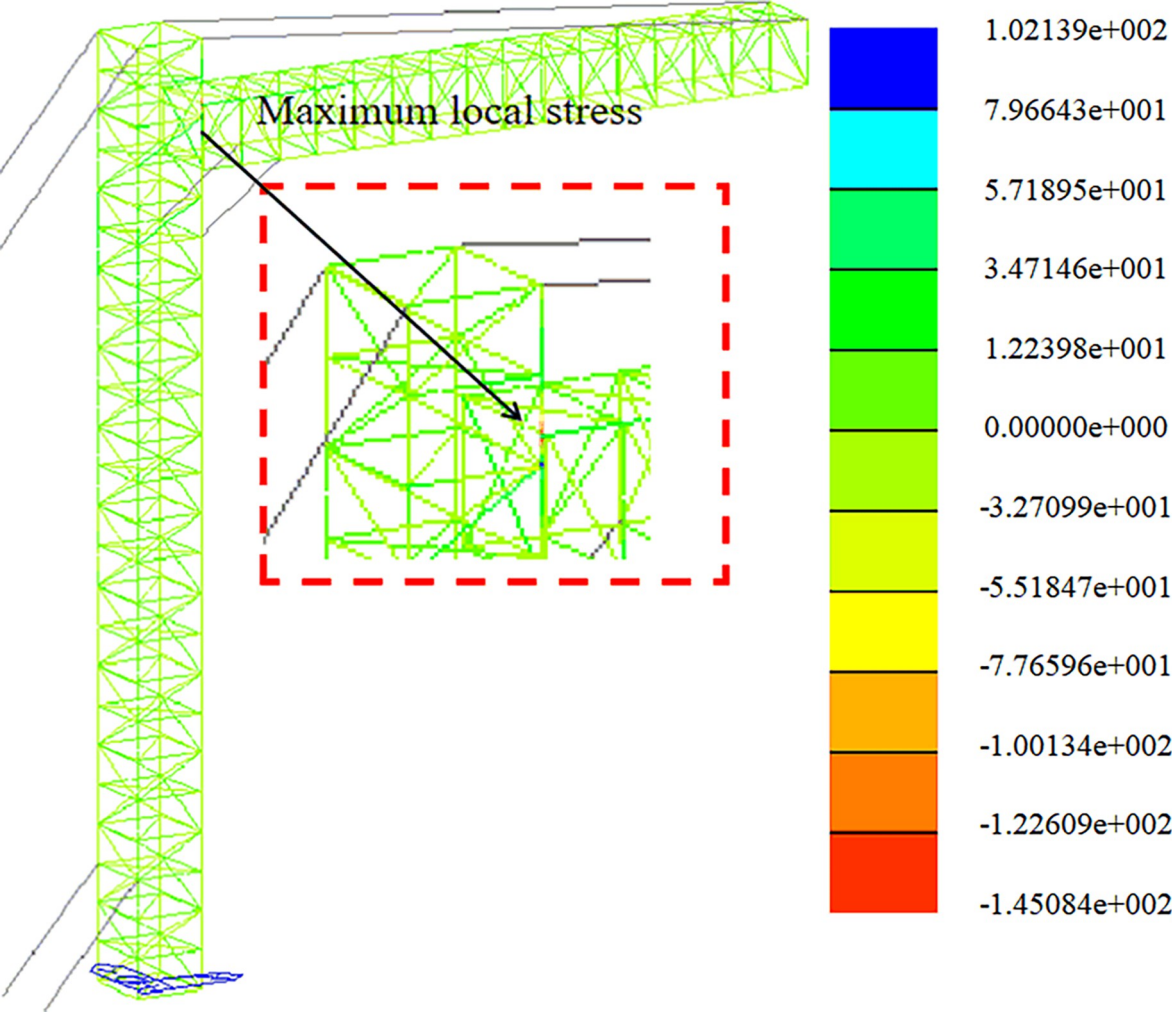
Stress nephogram under construction condition (+Y direction).

**Fig 10:**
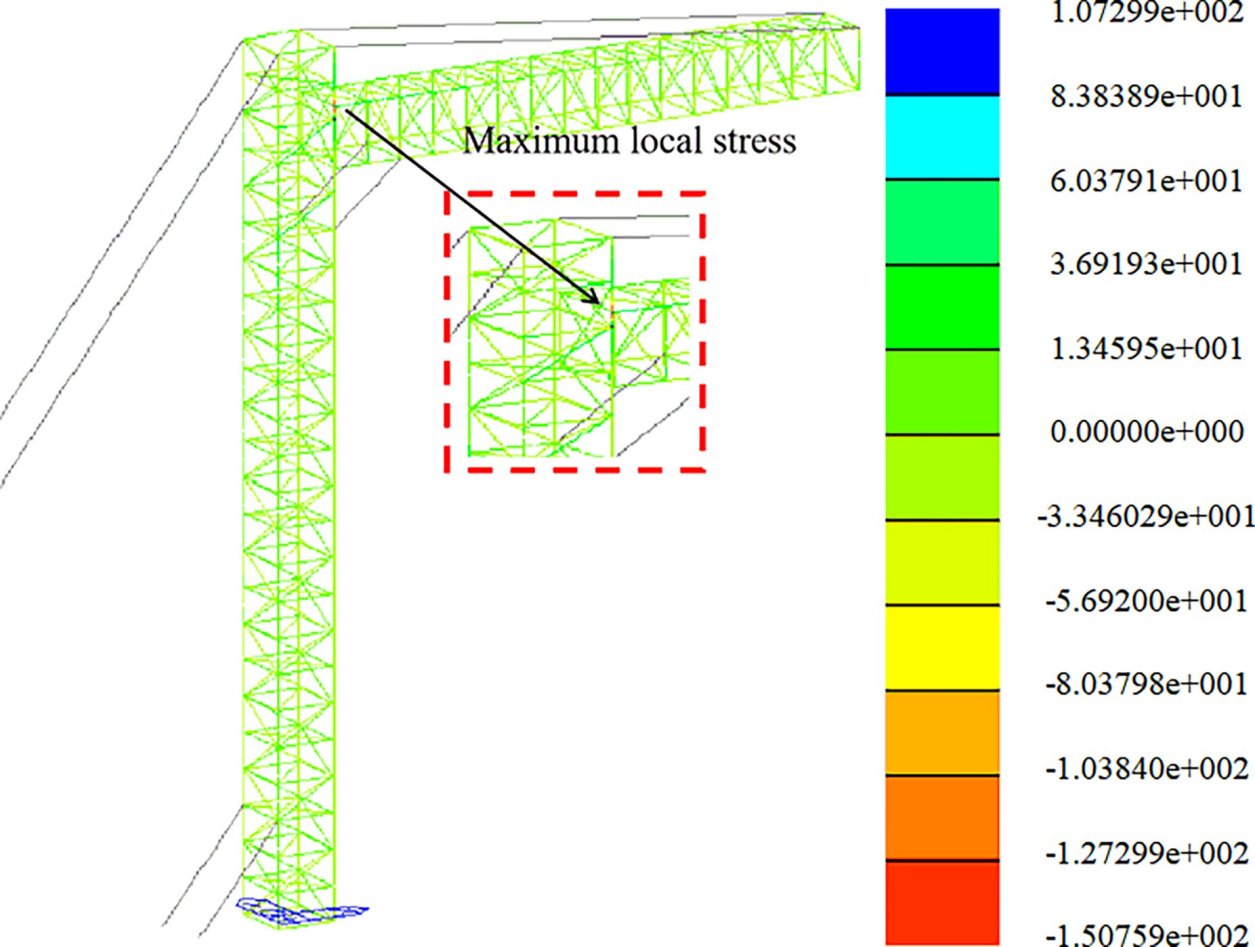
Stress nephogram under construction condition (45° direction).

Strength analysis of construction working conditions. The stress distribution in the +Y direction and at a 45-degree angle is depicted in Figs 9 and 10.

**Fig 11:**
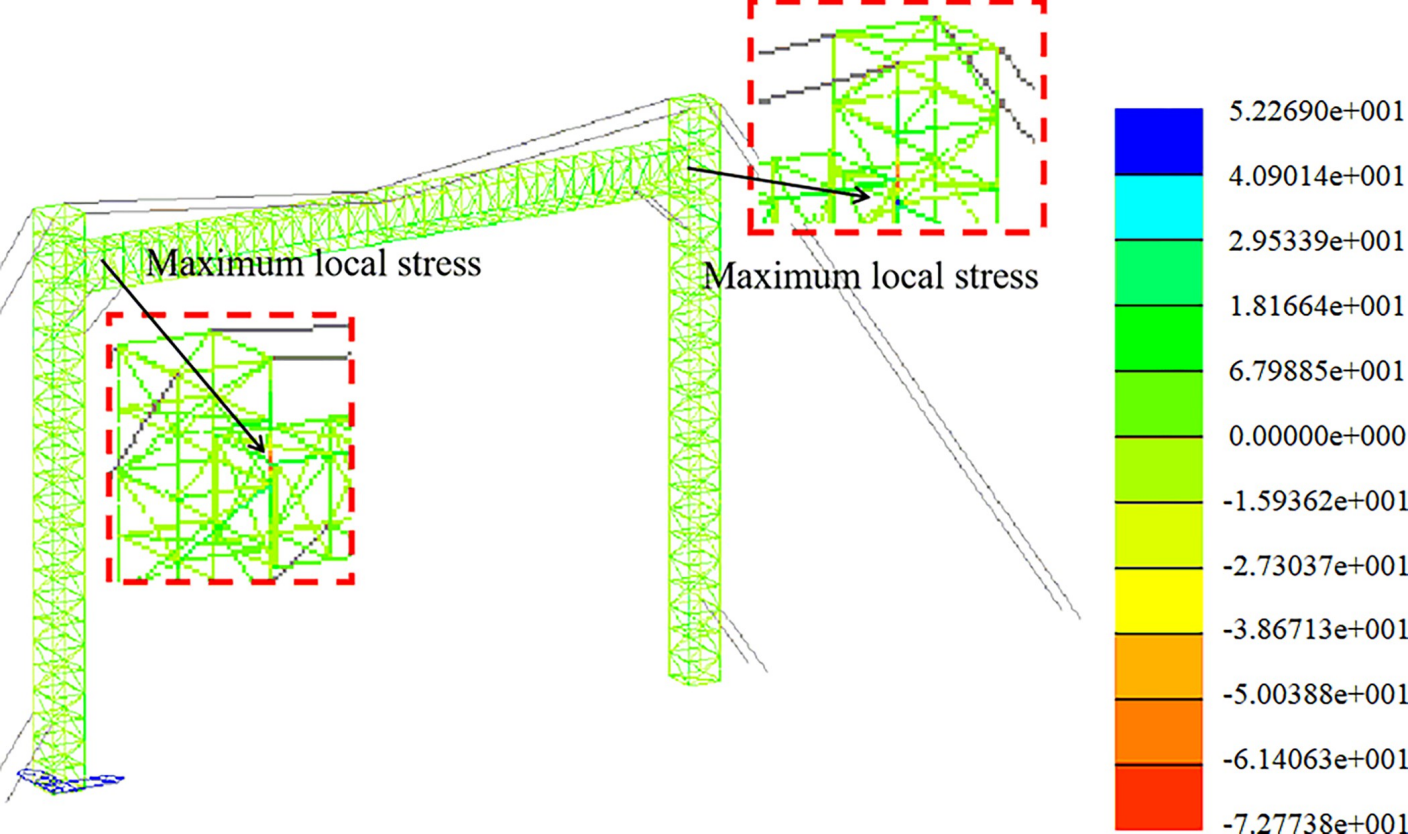
Stress nephogram under working condition (+Y direction).

**Fig 12:**
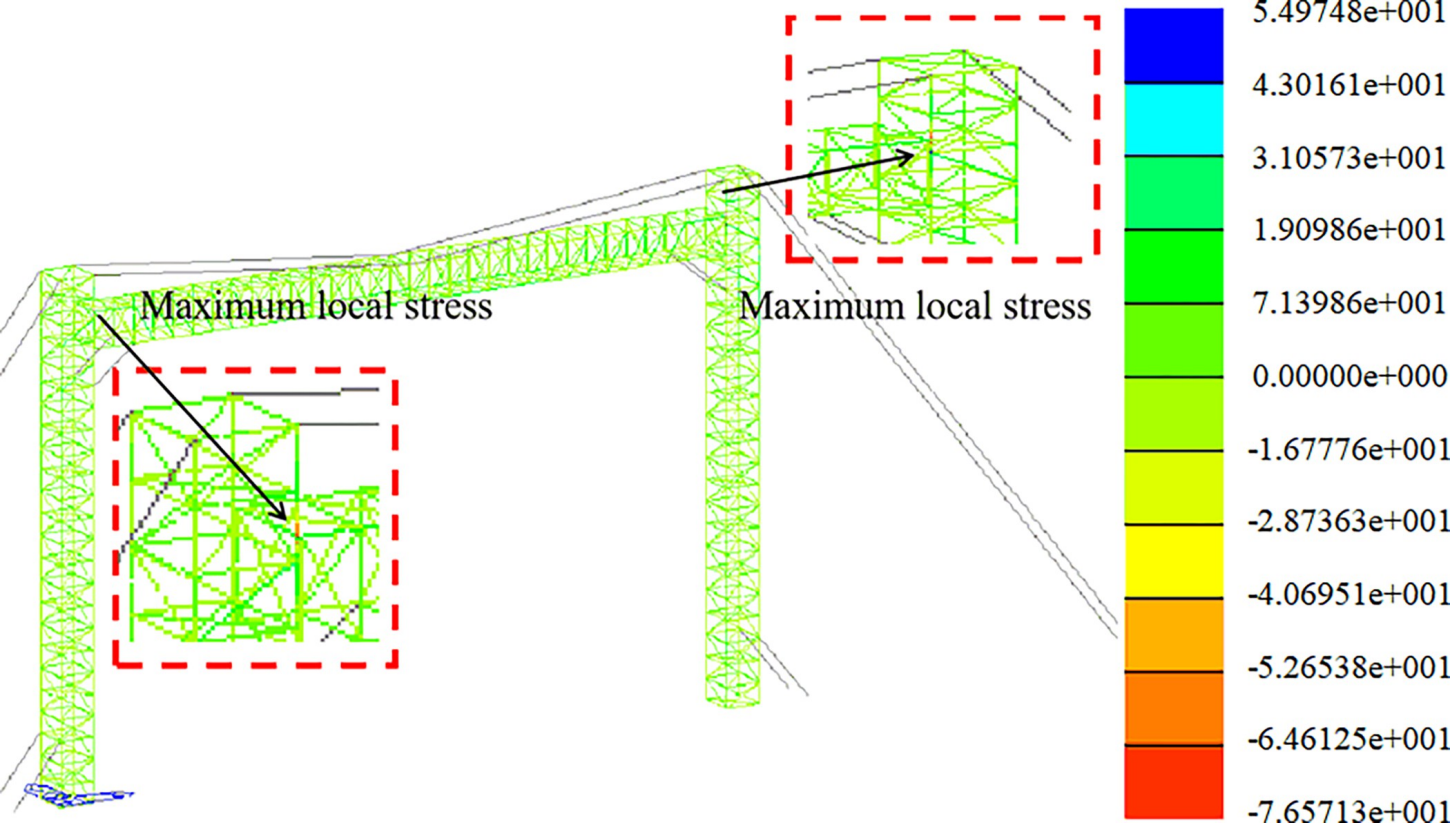
Stress nephogram under working condition (45° direction).

Strength analysis of working conditions. The stress distribution in the +Y direction and at a 45-degree angle is depicted in Figs 11 and 12.

**Fig 13:**
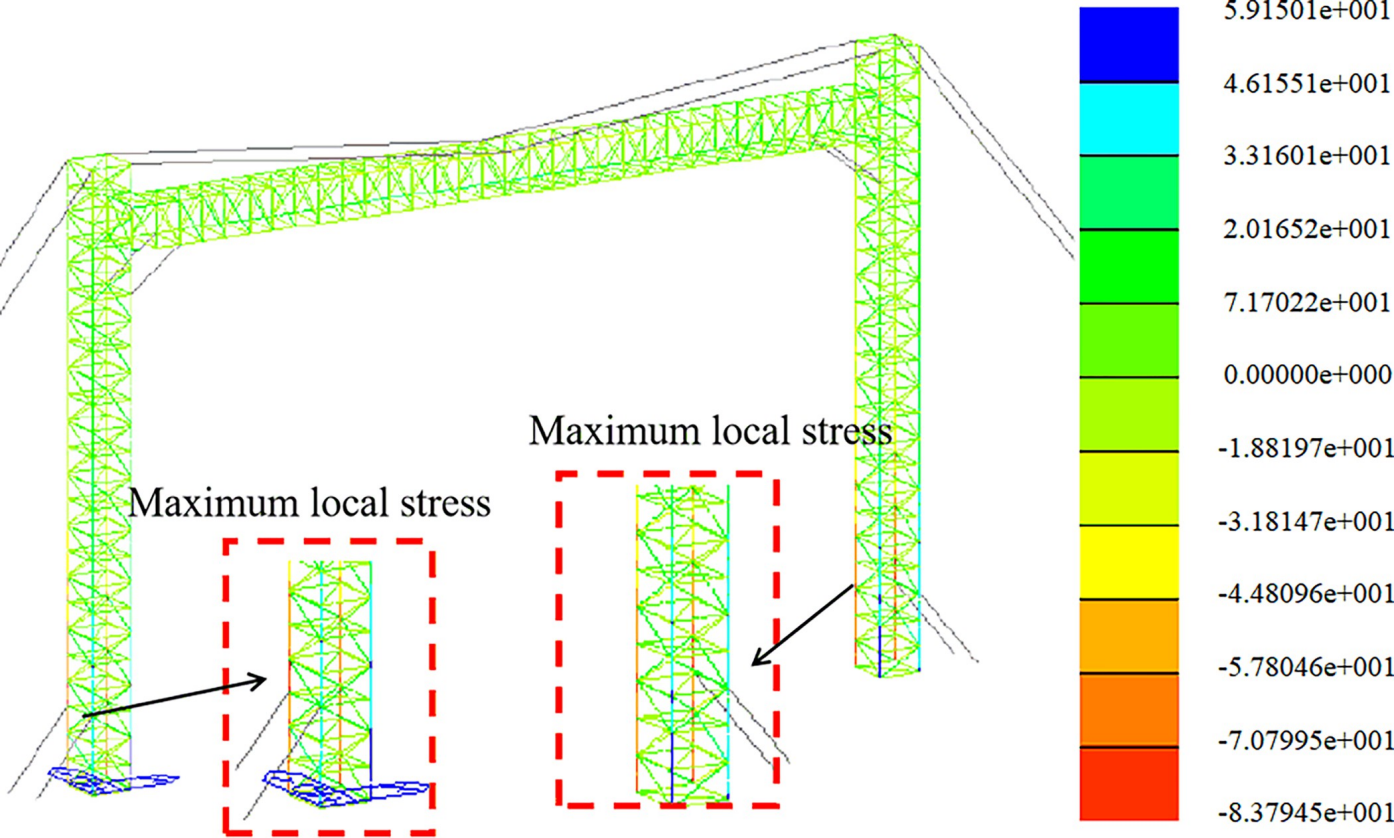
Stress nephogram under gale condition (+Y direction).

**Fig 14:**
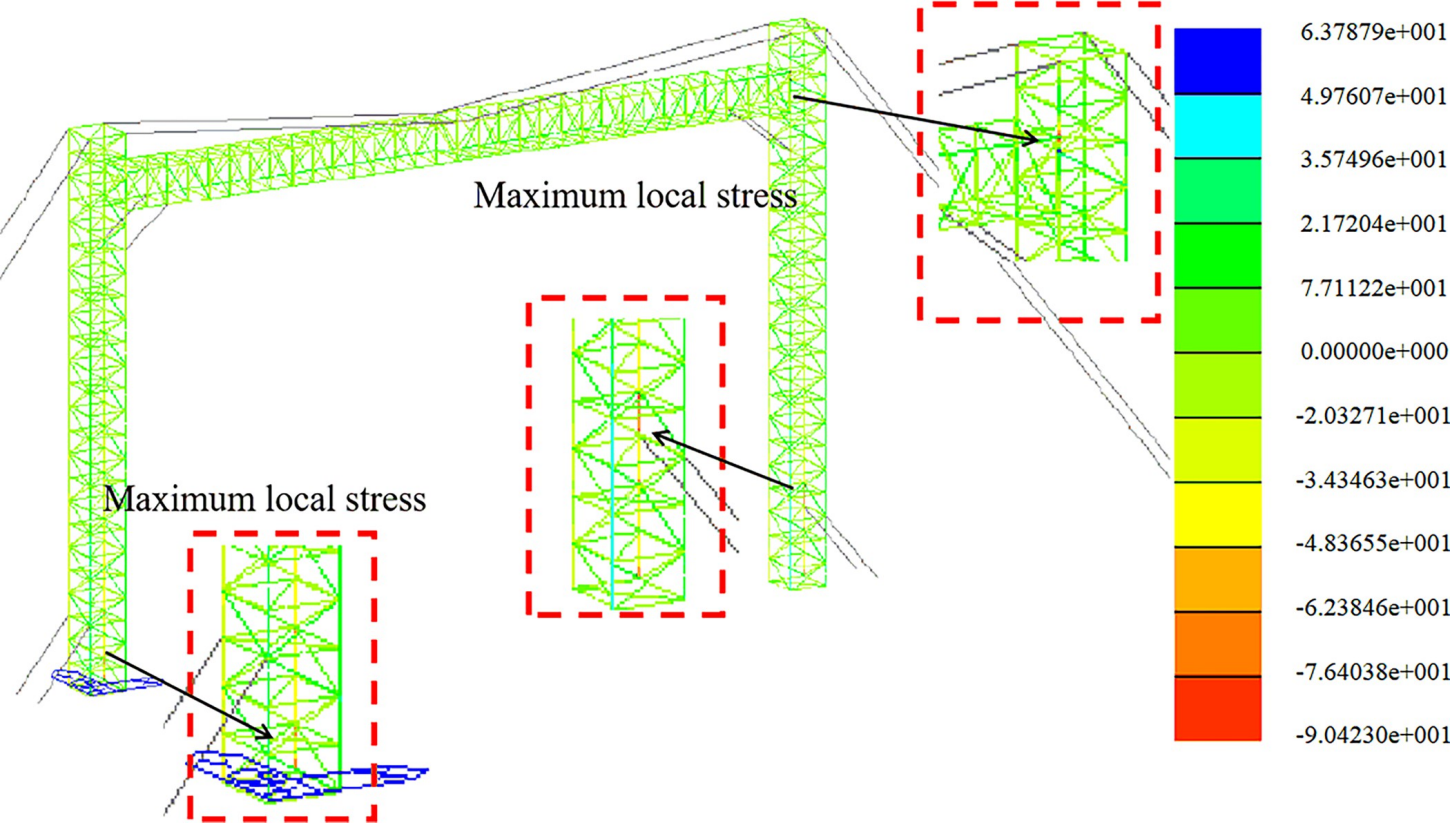
Stress nephogram under gale condition (45° direction).

Strength analysis of high wind conditions. The stress distribution in the +Y direction and at a 45-degree angle is depicted in Figs 13 and 14.

The maximum stress of the span frame structure under 9 working conditions is shown in Table 9., and it can be seen that the span frame structure under each working condition is in line with the allowed value of strength.

**Table 9:** Calculation and check of crossing frame stress.

Work conditions	1	2	3	4	5	6	7	8	9
**Maximum stress [Mpa]**	151.4	145.1	150.8	75	72.8	76.6	82.6	83.8	90.4

### Span frame stiffness analysis

The strength analysis of the optimized span frame model is carried out under the combined load action in each working condition. In this section, the results of the displacement calculations of the span frame in the construction assembly condition, the working condition of the line, and the extreme wind condition are given. The finite element analysis is carried out sequentially according to the load conditions and the working arrangement. The deformation diagrams and displacement clouds under each working condition are as follows.

**Fig 15:**
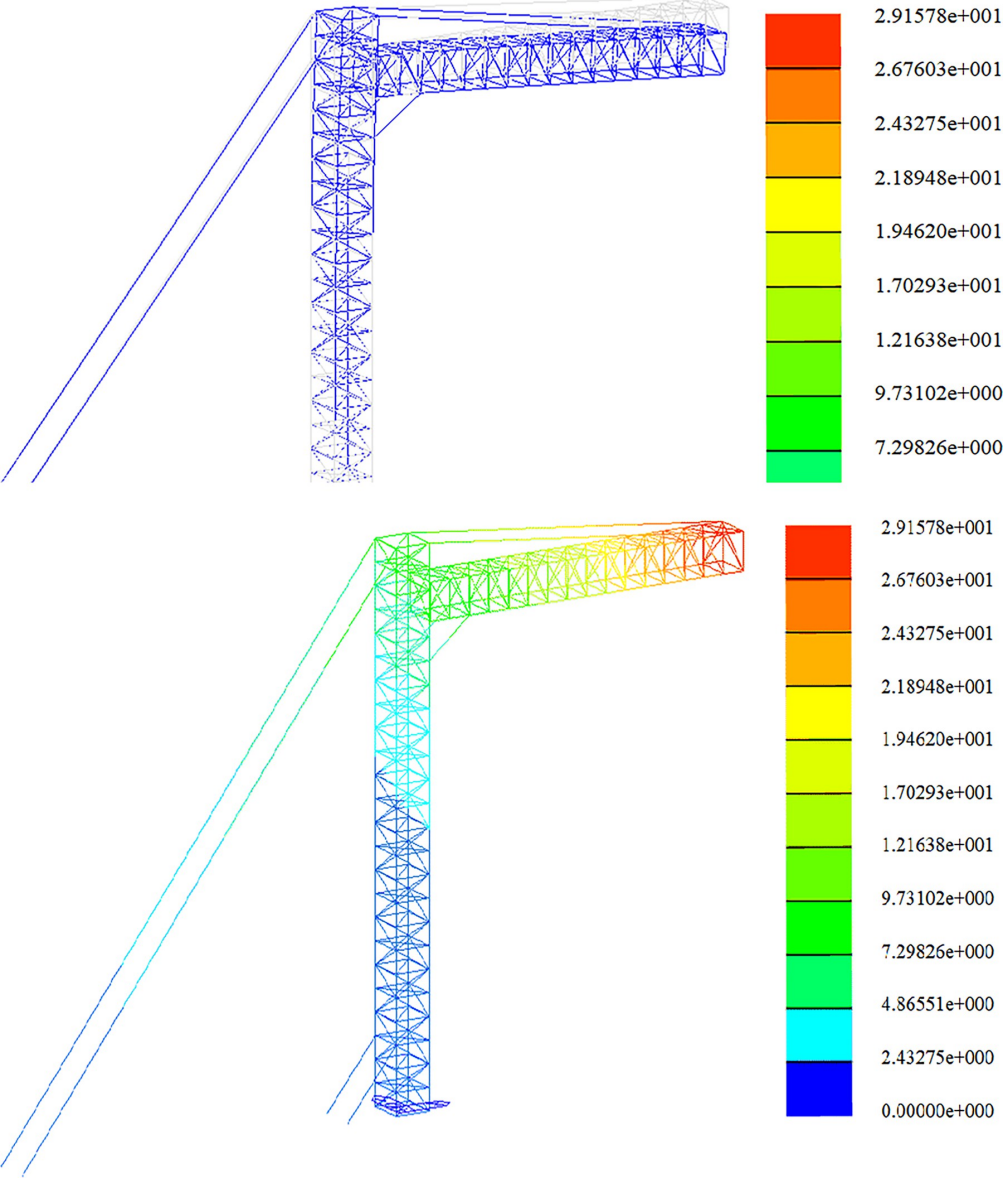
Nephogram of deformation and displacement under construction condition (+Y direction). Fig (a) Transformation chart. Fig (b) Displacement chart.

**Fig 16:**
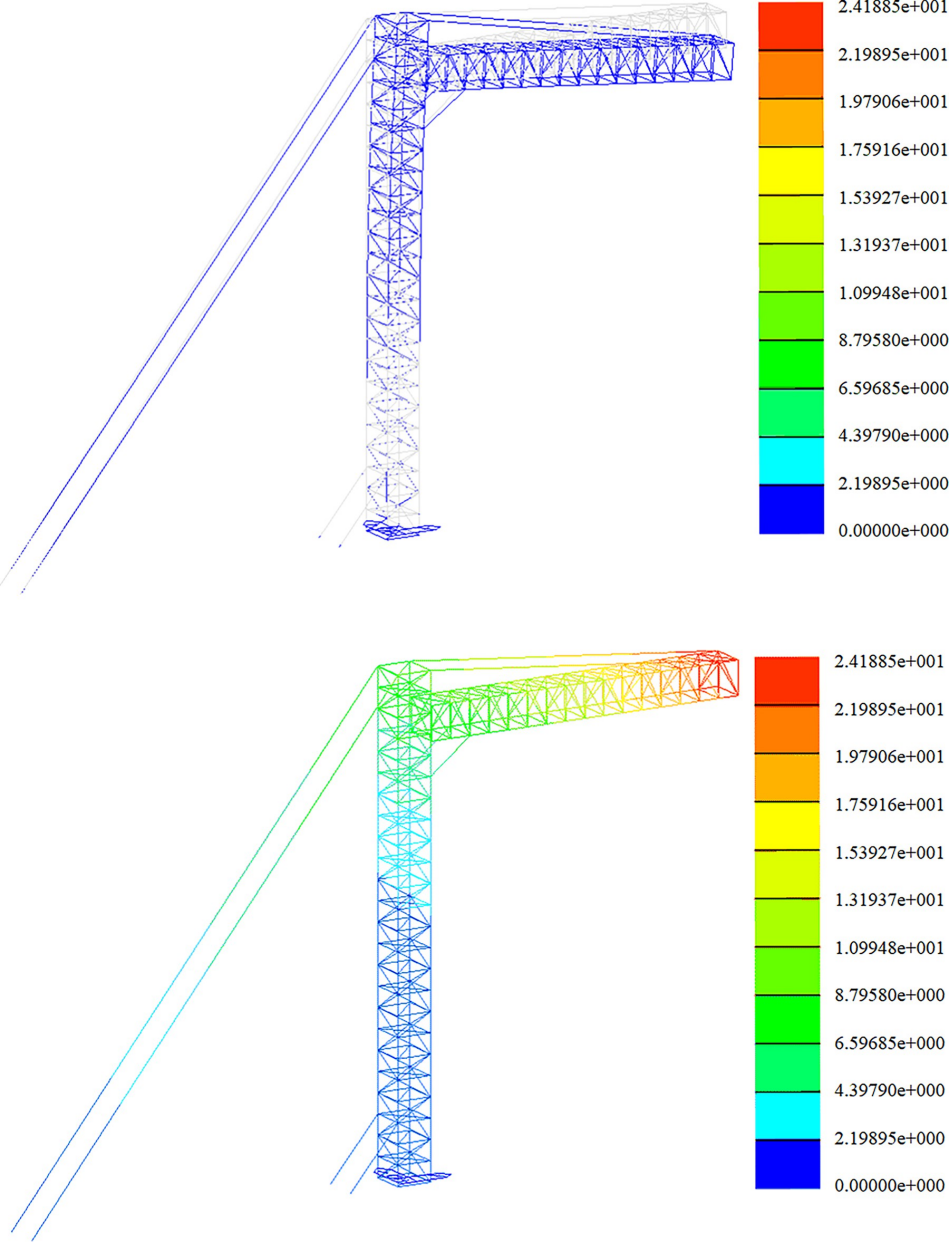
Nephogram of deformation and displacement under construction condition (45°direction). Fig (a) Transformation chart. Fig (b) Displacement chart.

**Fig 17:**
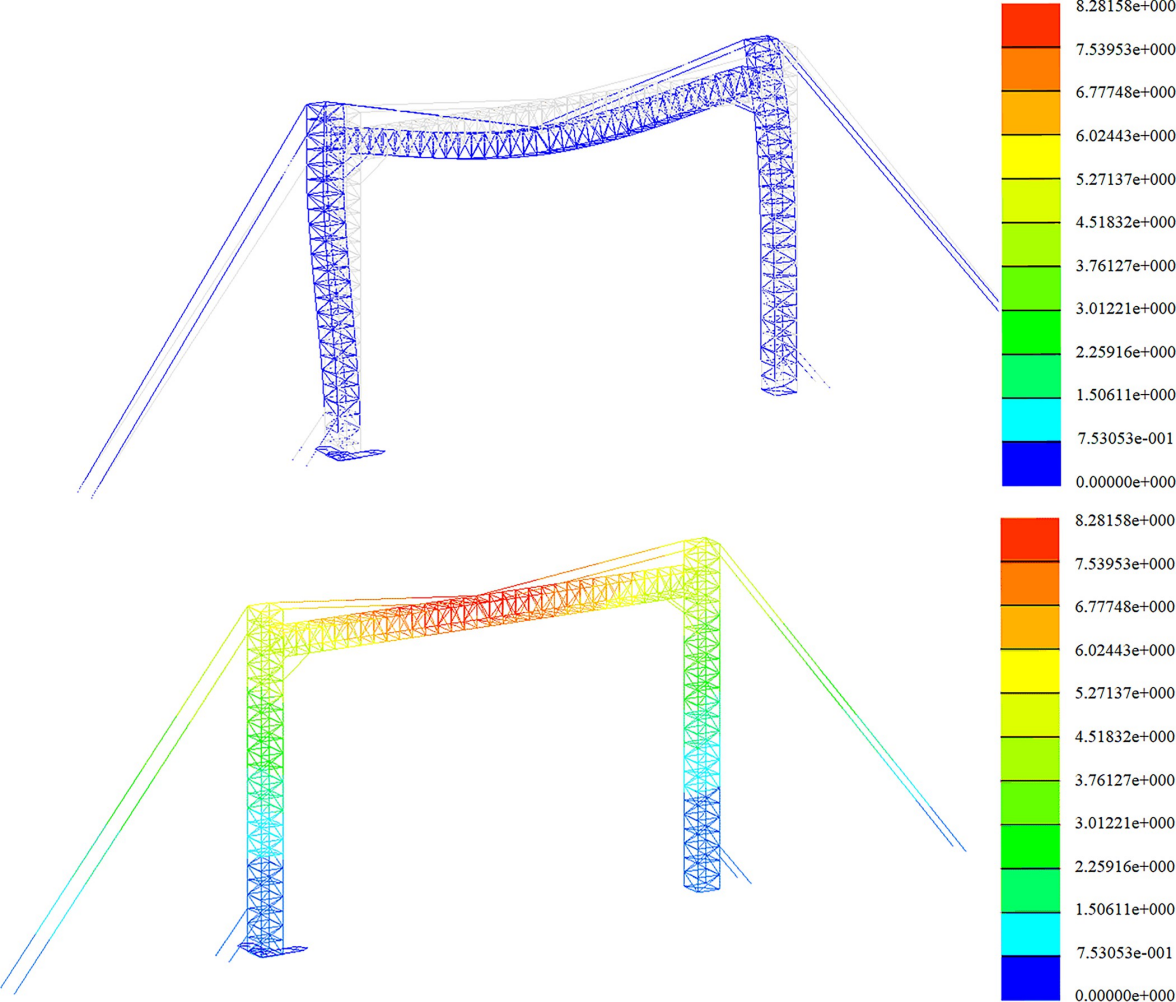
Working condition deformation and displacement nephogram (+Y direction). Fig (a) Transformation chart. Fig (b) Displacement chart.

**Fig 18:**
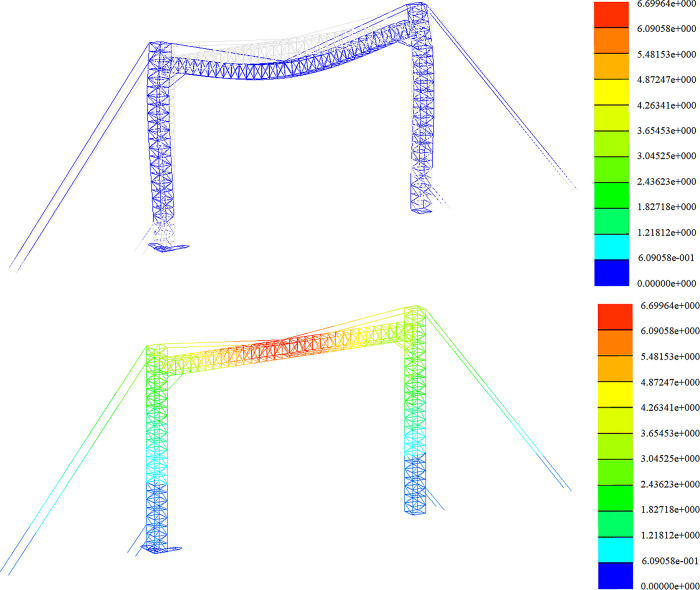
Working condition deformation and displacement nephogram (45°direction). Fig (a) Transformation chart. Fig (b) Displacement chart.

**Fig 19:**
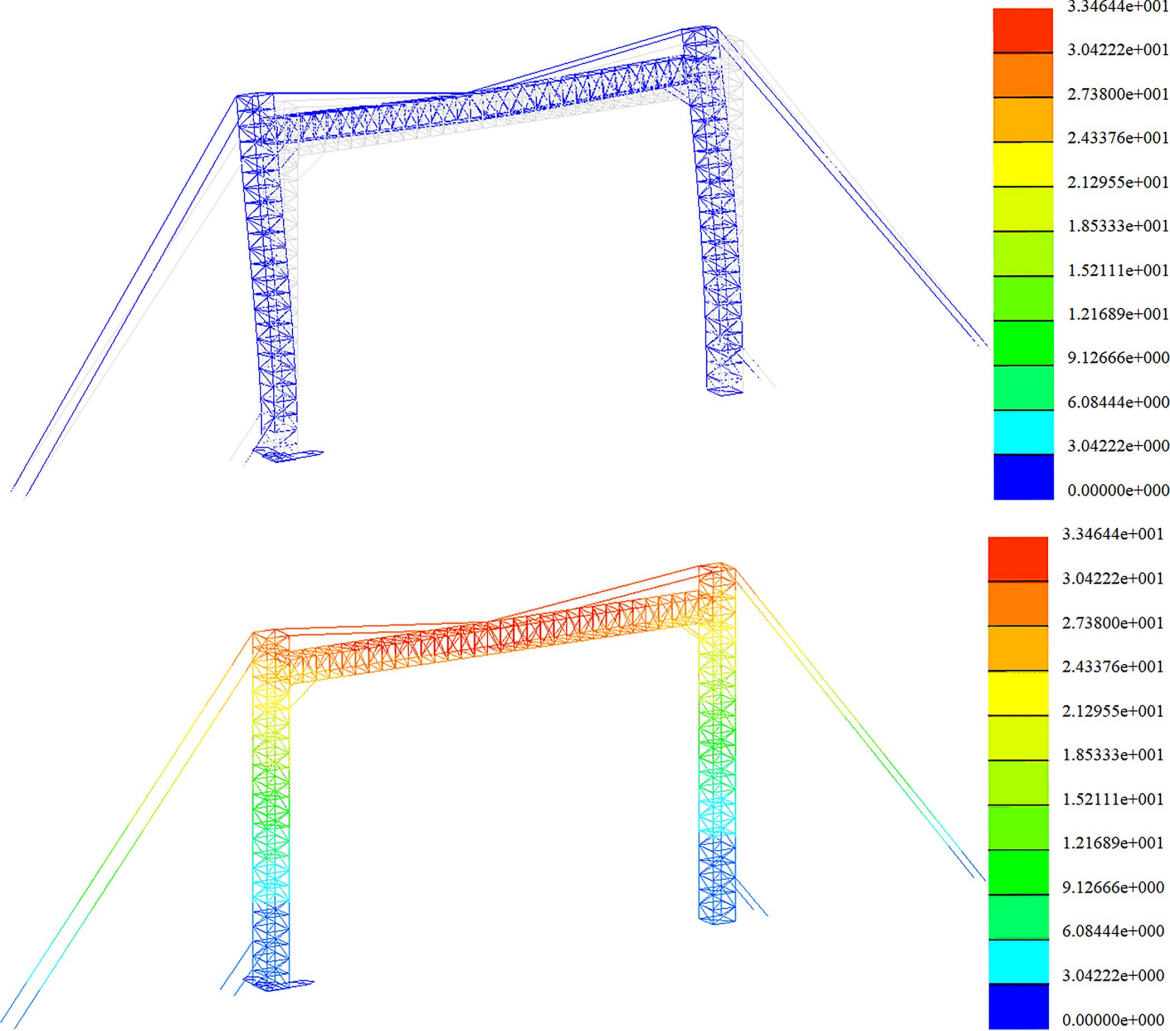
Diagram of deformation and displacement in gale condition (+Y direction). Fig (a) Transformation chart. Fig (b) Displacement chart.

**Fig 20:**
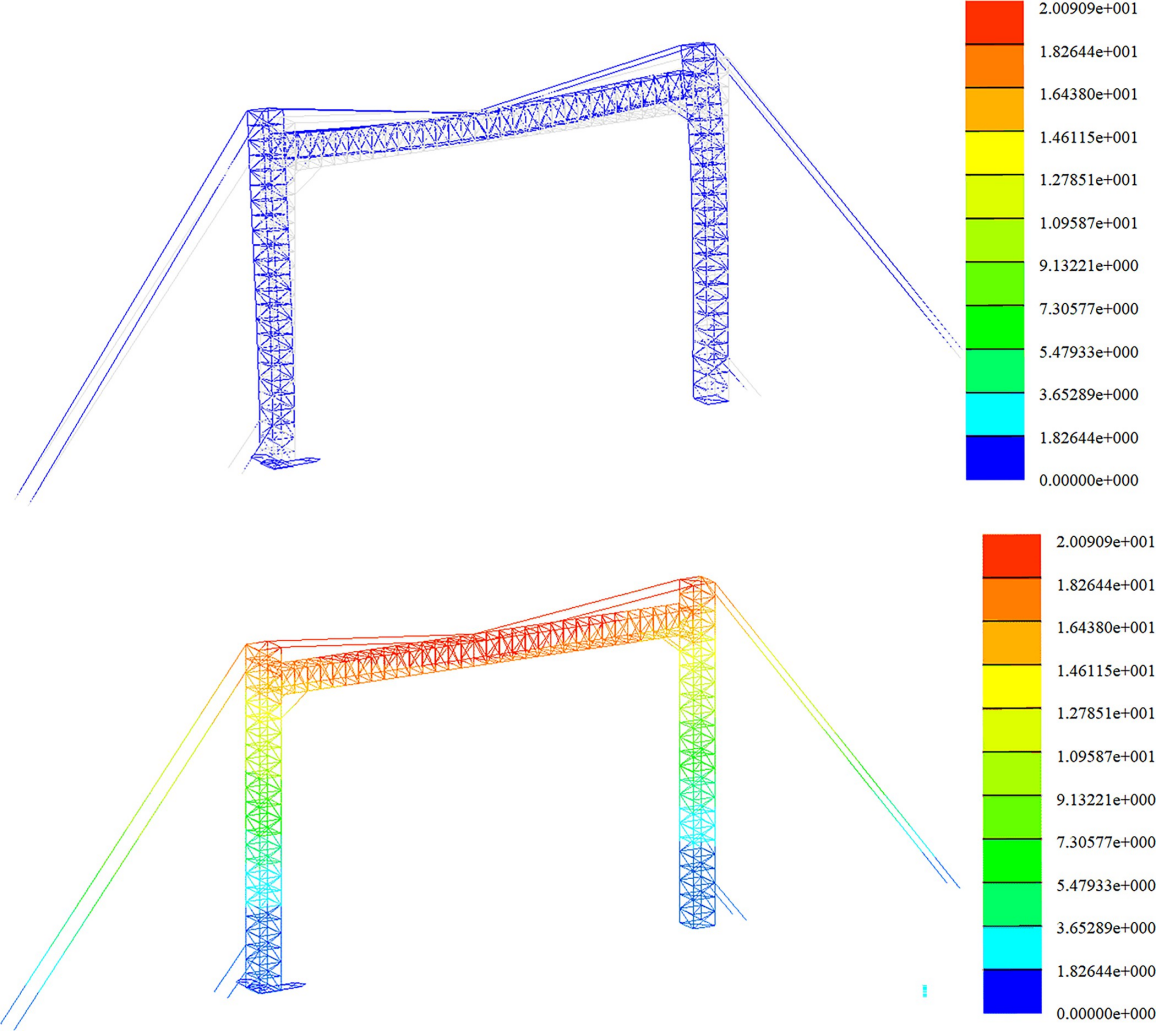
Deformation and displacement diagram under gale condition (45°direction). Fig (a) Transformation chart. Fig (b) Displacement chart.

Stiffness analysis of construction working conditions, as shown in Figs 15 and 16;

Stiffness analysis of working conditions, as shown in Figs 17 and 18;

Stiffness analysis of high wind conditions, as shown in Figs 19 and 20.

According to the design code, the relationship between the vertical static deflection Δ1 generated by the combined load at the cantilever position and the cantilever length L is:

$$\Delta_{1}\leq \frac{1}{350}L=\frac{1}{350}\times 12000mm=34.28mm$$ (10)

The relationship between the vertical static deflection Δ2 generated at the mid-span position and the crane span S is given by:

$$\Delta_{2}\leq \frac{1}{400}S=\frac{1}{400}\times 24000mm=60mm$$ (11)

The relationship between the static displacement Δ3 generated by the main column at the connection with its boom and the height H of the main column is:

$$\Delta_{3}\leq H\times 1.34\%=1.34\%\times 18000mm=241.2mm$$ (12)

Whether the structure of the span frame is safe and reasonable, the maximum displacement of the structural members under the action of each working condition should be checked to see whether it meets the requirements of the allowable value of the specification. When the height of the main column of the span frame is 18m, and the width of the boom is 12m, the stiffness calculation results are shown in Table 10., and it can be seen from Table 10. that the displacement of each position of the span frame meets the permitted value.

**Table 10:** Calculation and check of crossing frame displacement.

**Before docking**	**Work conditions**	**Jib end [mm]**	**Main column and jib connection [mm]**
	1	26.16	8.09
	2	26.76	8.31
	3	24.19	8.61
**After docking**	**Work conditions**	**Cross-center position [mm]**	**Main column and boom connection [mm]**
	4	5.54	1.12
	5	8.28	5.52
	6	6.70	3.59
	7	5.79	3.15
	8	33.46	28.73
	9	20.09	17.23

### Cross-frame stability analysis

Stability analysis is categorized into overall stability analysis and local stability analysis. The former is primarily considered when the span frame is subjected to a critical load, resulting in the loss of the structure’s original equilibrium. In contrast, local stability analysis focuses on the steel cross-sectional length ratio and the width-to-thickness ratio. This section delves into both the overall and local stability of the span frame.

It is crucial to account for structural deformation in the design and arrangement of towering structures, necessitating deformation verification. Linear buckling analysis provides structural critical load coefficients and identifies corresponding buckling modes for truss and beam units.

According to design standards, the critical buckling instability coefficient should be no less than 2. Buckling instability analysis, incorporating the span’s self-weight and load as variables, was conducted. The results, presented in Table 11, indicate that the buckling coefficients for each working condition satisfy the established requirements.

**Table 11:** Buckling analysis results.

Work conditions	Construction work conditions(+Y)	Working conditions(+Y)	High wind conditions(+Y)
**Buckling factor**	15.43	12.64	8.78

**Fig 21:**
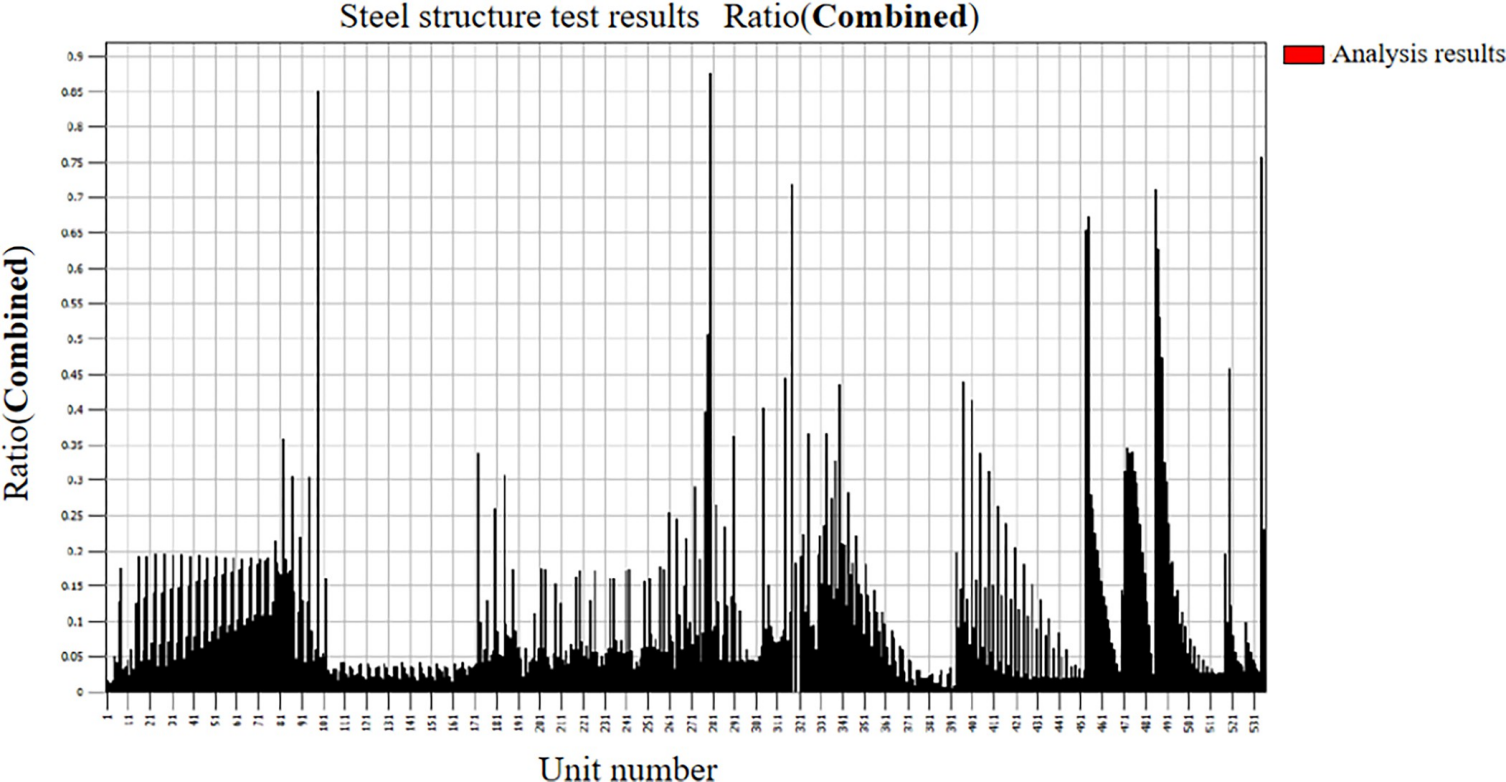
Ratio of checking calculation results of steel structure of crossing frame.

The results of the steel structure test are shown in Fig 21. Under each working condition, the cross-sectional test results of the span frame structure bars are shown in Table 12. The maximum stress ratio of the bar with cross-sectional number 3, the main column diagonal web bar, is 0.88. The bar is mainly subject to axial force, which is in accordance with the stress characteristics of the truss structure, and the strength and stability of the single bar meet the requirements. And the stress ratio of each rod is close to the allowable stress value, which indicates that the optimized rod has good utilization rate.

**Table 12:** Section checking table.

Work conditions	Rod number	Cross-sectional parameters	Control conditions	Axial force [N]	Bending moment[N∙m]	Stress ratio
1	L63×63×5.0	5	8290.82	3793.8	0.85	1
2	L56×56×5.0	6	-18929	-55.82	0.34	2
3	L40×40×4.0	4	-30789	-40.62	0.88	3
4	L36×36×5.0	7	-4570.1	-17.09	0.44	4
5	L45×45×6.0	6	38105.3	370.45	0.72	5
6	L45×45×3.0	9	-9534.8	18.42	0.44	6
7	L36×36×4.0	7	-8927.6	9.09	0.71	7
8	L36×36×3.0	5	-3237.3	-6.51	0.46	8

## Discussions

### Comparison with existing methods

The optimization approach detailed in this study leverages the BBO algorithm’s unique capabilities, notably its effectiveness in addressing complex optimization problems. When juxtaposed with conventional methods, such as those cited in the introduction (e.g., genetic algorithms, probabilistic natural selection, and other meta-heuristic algorithms), the BBO algorithm demonstrates superior performance in optimizing the size, shape, and topology of transmission type frame structures.

The results indicate that the BBO algorithm, when applied to the optimization of transmission line spanning frames, yields a structure that is not only lighter but also satisfies all predefined constraints related to strength, stiffness, and stability. This outcome underscores the algorithm’s efficiency in navigating the design space to identify optimal solutions that might be elusive for other optimization techniques.

### Practical implications

The study’s findings have significant practical implications. The optimized frame structures, which are lighter yet robust, can lead to cost savings in material and construction. Furthermore, the method’s ability to adhere to various engineering constraints ensures that the optimized structures are not only economically beneficial but also meet the stringent requirements of real-world engineering applications.

The integration of the BBO algorithm with finite element analysis tools, as demonstrated in this study, offers a powerful approach for engineers and designers. This integrated method can be applied to a wide range of optimization problems in civil and structural engineering, beyond just transmission line frames, indicating its versatility and broad applicability.

### Limitations and future work

While the BBO algorithm has shown promising results, it is not without its limitations. The convergence rate and the algorithm’s sensitivity to initial parameters are areas that require further investigation. Future research could explore modifications or hybridizations of the BBO algorithm to enhance its performance, especially in handling constraints and improving convergence rates.

Moreover, comparative studies with other advanced optimization algorithms could provide deeper insights into the strengths and weaknesses of various approaches in the context of structural optimization. Such comparative analyses could guide the selection of the most appropriate optimization tool based on specific design requirements and constraints.

## Conclusions

This study introduces a comprehensive optimization design method for transmission line crossing frame structures based on the Biogeography-Based Optimization (BBO) algorithm. Integrating size, shape, and topology optimization, this research not only enhances the frame structures’ performance but also ensures the economic and practical aspects of the structural design. The integrated design method produces a frame structure that is lighter and has an acceptable maximum stress of 151.4 MPa compared to separate optimizations for size or shape and topology, validating the BBO algorithm’s effectiveness and practicality in addressing complex structural optimization challenges.

The application results of the BBO algorithm indicate a 15% reduction in the structural mass of the transmission line crossing frame and a 30% decrease in the number of iterations required during the optimization process. Compared to conventional optimization methods, the approach based on the BBO algorithm demonstrates superior efficiency and effectiveness in exploring design variables and identifying global optimal solutions. The method’s validity and applicability are further corroborated through finite element analysis of the optimized frame, reinforcing its efficacy and suitability.

Additionally, the study proposes a comprehensive optimization scheme that includes discrete and logical topology variables, enabling the retention of different types of design variables for global optimized design. By employing a logical judgment form to predefine the bar arrangement method that meets actual engineering and specification requirements, the research achieves synchronized optimization of size, shape, and topology.

The final analysis, conducted under established working conditions in accordance with specifications, confirms that the optimized structure meets all design constraints, including strength, stiffness, and stability requirements.

This research not only offers a new perspective and method for the optimization design of transmission line crossing frames but also highlights the BBO algorithm’s potential and value in the field of engineering structural optimization design. Future work will focus on enhancing the algorithm’s convergence speed and optimization efficiency and exploring its application in a broader range of engineering structural optimization design problems.
